# Metabolomic homeostasis shifts after callus formation and shoot regeneration in tomato

**DOI:** 10.1371/journal.pone.0176978

**Published:** 2017-05-08

**Authors:** Alka Kumari, Kamalika Ray, Sadhna Sadhna, Arun Kumar Pandey, Yellamaraju Sreelakshmi, Rameshwar Sharma

**Affiliations:** Repository of Tomato Genomics Resources, Department of Plant Sciences, University of Hyderabad, Hyderabad, India; Austrian Federal Research Centre for Forests BFW, AUSTRIA

## Abstract

Plants can regenerate from a variety of tissues on culturing in appropriate media. However, the metabolic shifts involved in callus formation and shoot regeneration are largely unknown. The metabolic profiles of callus generated from tomato (*Solanum lycopersicum*) cotyledons and that of shoot regenerated from callus were compared with the *pct1-2* mutant that exhibits enhanced polar auxin transport and the *shr* mutant that exhibits elevated nitric oxide levels. The transformation from cotyledon to callus involved a major shift in metabolite profiles with denser metabolic networks in the callus. In contrast, the transformation from callus to shoot involved minor changes in the networks. The metabolic networks in *pct1-2* and *shr* mutants were distinct from wild type and were rewired with shifts in endogenous hormones and metabolite interactions. The callus formation was accompanied by a reduction in the levels of metabolites involved in cell wall lignification and cellular immunity. On the contrary, the levels of monoamines were upregulated in the callus and regenerated shoot. The callus formation and shoot regeneration were accompanied by an increase in salicylic acid in wild type and mutants. The transformation to the callus and also to the shoot downregulated *LST8* and upregulated *TOR* transcript levels indicating a putative linkage between metabolic shift and TOR signalling pathway. The network analysis indicates that shift in metabolite profiles during callus formation and shoot regeneration is governed by a complex interaction between metabolites and endogenous hormones.

## Introduction

One distinctive character that distinguishes plants from animals is their remarkable capacity for regeneration. The differentiated mature plant cells can undergo dedifferentiation followed by organogenesis and generation of fully fertile plant. On injury of organs, cells in the vicinity of the injury proliferate to form a mass of soft tissue consisting of pluripotent cells called as callus to seal the injured area. During last three decades, *in vitro* initiation of callus and organogenesis has been used for a plethora of biotechnological applications. Though Haberlandt (1902) [1] proposed the idea of *in vitro* culturing of isolated cells/tissues from higher plants, the successful tissue culture could be established only after supplementation of media with plant hormone indoleacetic acid (IAA) [2], which was identified few years earlier [3]. The discovery of another plant hormone cytokinin and realization that the initiation of shoot or root from callus can be induced by alteration of ratios of cytokinin to IAA [4] stimulated regeneration of several plant species using tissue culture. The observation that a single cell from carrot root cells suspension culture can differentiate into a somatic embryo provided conclusive proof that plant cells are totipotent in nature [5]

In recent years, the molecular basis underlying dedifferentiation to callus and also for initiation of organogenesis has been uncovered. Detailed examination of calli initiation in Arabidopsis revealed that incubation of shoot/root explants on auxin rich callus initiating media (CIM) triggers initiation of callus from the pericycle cells in vascular bundles [6]. Also, the cells constituting calli were not a mass of undifferentiated cells rather had organized structures akin to the primordia of lateral roots [6]. Even the calli initiated from other organs such as cotyledons and petals showed organized structures similar to lateral root primordia [7]. The notion that the molecular events leading to calli initiation were similar to initiation of lateral root primordia was supported by the resemblance of transcriptome profiles of calli to that of lateral root primordia [7]. The suppression of calli initiation in *aberrant lateral root formation4* (*alf4*) mutant also supported that, at least in Arabidopsis, the calli initiation follows a pathway similar to initiation of lateral roots [7].

The initiation of calli requires reacquisition of mitotic cell division which is stimulated by the exogenous plant hormones. Among hormones, auxin is a well-known inducer of lateral root development. It is reported that during callus initiation auxin activates ARF7 and ARF19 transcription factors that in turn activate LBD transcription factors [8], [9]. The induction of cell cycle regulatory E2Fa transcription factor by LBD transcription factor stimulates re-entry into S phase of cell cycle [10]. Since lateral root formation is impeded in *E2Fa* mutant, it supports the operation of ARF-LBD-E2Fa pathway for calli initiation [9]. In parallel, auxin also stimulates callus induction by downregulation of *KRP* genes relieving inhibition of CDKs stimulating cell cycle progression [11]. Compared to auxin, limited information is available about the role of cytokinin in callus initiation. It is reported that cytokinin may mediate its action by regulating the activity of type-B ARR, AP2/ERF, ESR1, and ESR 2 transcription factors [12].

Currently, it is believed that the callus basically has a root identity [13]. The transfer of callus to cytokinin rich shoot initiation media (SIM) triggers switch from root to shoot identity with induction of shoot-specific genes such as *WUS* and *CUC2* [14]. On SIM, calli also show the segregation of gene expression pattern, with auxin-responsive genes being confined to the region not initiating shoot, and cytokinin responsive genes being confined to region initiating shoot [14], [6]. Also, both calli induction and shoot induction is regulated by changes in the levels of microRNA. In Arabidopsis calli grown on SIM, *MIR160a* levels were more downregulated in totipotent calli than in non-totipotent calli [15]. It is recently reported that the initiation of the calli and regeneration of shoot occurs via a two-step mechanism. In first step *PLETHORA* genes (*PLT3*, *PLT5*, and *PLT7*) control the expression of root stem cell maintenance regulators (*PLT1* and *PLT2*) leading to calli initiation and proliferation. In second step plethora genes (*PLT3*, *PLT5*, and *PLT7*) regulate initiation of shoot regeneration by modulating shoot-promoting factors (CUC2) [16].

While the gene regulation is foremost in driving the developmental transitions, these transitions are also coupled with changes in the levels of primary metabolites and phytohormones. Though it is known that exogenously added hormones, partially exert their effect by altering endogenous hormone levels, very few studies investigated alteration in endogenous hormone levels during callus induction and organogenesis. The regeneration ability of pumpkin cotyledons varied depending on the endogenous cytokinin (iPA) content of different aged seedlings [17]. In rice, exposure to osmotic stress elevates the levels of endogenous abscisic acid (ABA) and IAA promoting shoot regeneration from callus [18].

Despite considerable research on the molecular-genetic basis of tissue regeneration, very few reports examined the metabolome profiles and its regulation during callus formation and shoot regeneration. In rice, profiling of callus, leaf, and panicle revealed distinct differences in metabolic profiles of respective tissue [19]. In sugar cane, leaf-derived embryogenic and non-embryogenic callus showed distinct metabolic profiles with higher levels of sugars such as glucose, fructose, sucrose, and maltose in embryogenic calli along with upregulation of few amino acids [20]. In recent years, evidences have also emerged that regulation of cellular metabolism in plants is mediated by TOR (Target of Rapamycin) signalling pathway in a fashion similar to animal and yeast cells [21], [22]. In Arabidopsis TOR interacts with RAPTOR and LST8 homologs to activate ribosomal protein S6 kinase (S6K), a regulator that promotes cell expansion [23]. Correlation with TOR activity and cellular metabolism has been observed in Arabidopsis *tor* mutant that shows smaller cells and organs similar to the plants growing under limited nutrient availability or low light irradiance [24], [25].

In this study, we examined the shift in metabolite profiles during *in vitro* organogenesis in tomato by comparing metabolite and hormonal profiles of cotyledon with callus and regenerated shoots. It is reported that PIN1 protein involved in polar auxin transport plays an important role during early stages of callus initiation displaying upregulation followed by downregulation in the region destined to be shoot progenitor cells [14], [6]. During shoot regeneration from calli, PIN1 is accumulated in developing shoot meristem and later in organ primordia. Consistent with this shoot regeneration in Arabidopsis *pin1-4* mutant is reduced to 1/5 of wild-type [14]. In view of above role of PIN1 in calli formation, we compared metabolic shifts with the polycotyledon (*pct1-2*) mutant of tomato that shows enhanced polar auxin transport associated with elevated level of polar auxin transporter PIN1 protein [26], [27].

Nitric oxide (NO) is an important regulatory molecule participating in a large number of developmental responses in the plants [28]. In tomato, NO participates in auxin-induced lateral root initiation as a part of signal transduction chain [29]. Evidences indicate that during tomato lateral root initiation NO modulates cell cycle regulatory genes mediating transition from G_1_-to-S phase [30]. Considering that callus has identity akin to lateral root primordia, and NO participates in lateral root initiation, we also compared the shifts in metabolite profiles with the shifts in tomato *short root* mutant where shortening of the root is associated with overproduction of nitric oxide [31].

We mapped the alteration of metabolites and endogenous hormones on respective metabolic pathways in an attempt to understand modulation of metabolomic homeostasis during callus formation and shoot regeneration. In addition to comparing relative levels of individual metabolites and hormones, we also used the network approach to obtain an integrated view of metabolite-metabolite and hormone-metabolite correlations [32]. We show that though the metabolic networks in *pct1-2* and *shr* mutants were distinct from wild type, the transition to callusing and organogenesis proceeded normally with shifts in endogenous hormones and metabolite interactions. Our results are discussed in the context of the transformation of cotyledon to callus, and callus to regenerated shoot on metabolite profiles and interaction networks. We also report that callus shows reduced lignification and lowered cellular immunity that may have a bearing on metabolomic homeostasis. In addition, we also report that callus formation and shoot regeneration alter transcript levels of genes mediating TOR signalling pathway.

## Materials and methods

### Plant material and growth conditions

The seeds of wild type (WT) tomato (*Solanum lycopersicum*) cv. Ailsa Craig, and *pct1-2* (enhanced polar auxin transport mutant, [26] and *shr* (NO overproducing mutant) [31] were used. Both *shr* and *pct1-2* mutants were monogenic recessive in nature. Seeds were surface sterilized with 4% (v/v) sodium hypochlorite, and seedlings were raised on MS (1/2X) medium [33] supplemented with 1.5% (w/v) sucrose under 16 h light and 8 h darkness cycles. Fully expanded cotyledons from 9–10 days old seedlings were used as explant for the callus initiation. For callus induction, excised cotyledons were cut at the tip and the base (0.7×1.0 cm size) and were placed on MS medium (1X) supplemented with 3% (w/v) sucrose, 2 mg l^-1^ zeatin and 0.1 mg l^-1^ IAA (CIM) with their adaxial surface in contact with the medium. Explants were sub-cultured at an interval of 10–12 days by transferring to fresh plates on CIM. After the appearance of shoots, the shoots were excised from the callus and transferred to the SIM consisting of MS media supplemented with 3% (w/v) sucrose, 1 mg l^-1^ zeatin and 0.1 mg l^-1^ IAA for further differentiation and growth.

For metabolite and hormone profiling, tissues were collected at three different stages; 9–10 day old trimmed cotyledon, greening callus and regenerated shoot. The tissue was snap frozen and homogenized in liquid nitrogen and stored at -80°C till further use. All experimental analyses with GC-MS or LC-MS were carried out using 5 independent biological replicates at each stage from WT, *shr* and *pct1-2*.

### GC-MS analysis

The GC-MS based identification of primary metabolites was carried out essentially using the protocol described by Roessner et al. (2000) [34]. A polar metabolite fraction was extracted from 100 mg of powdered tissue by shaking with 1400 μl 100% methanol and 60 μl internal standard (0.2 mg ml^-1^ ribitol in MilliQ water) at 700 rpm for 15 min at 70°C. The extract was vigorously mixed with 1400 μl water, centrifuged at 2200*g* at 25°C and supernatant was collected. 300 μl aliquots of supernatant were dried *in vacuo* for 3 h. The dried residue was dissolved and derivatized for 90 min at 37°C in 80 μl of pyridine containing 20 mg ml^-1^ methoxyamine hydrochloride. To the derivatized sample 80 μl N-trimethylsilyl-N-methyl trifluoroacetamide (MSTFA) and 20 μl of a retention time standard mixture (F.A.M.E. Mix, Sigma, 1 μg μl^-1^ in hexane) was added, and the mixture was incubated at 37°C for 30 min.

The derivatized samples were then analysed by LECO-PEGASUS GCXGC-TOF-MS system (LECO Corporation, USA) equipped with 30 m Rxi-5ms column with 0.25 mm internal diameter and 0.25 μm film thickness (Restek, USA). The injection temperature, interface, and ion source were set at 250°C, 225°C and 200°C respectively. For separation of groups of metabolites, the run program was set as following; isothermal heating at 70°C for 5 min, followed by 5°C min^−1^ oven temperature ramp to 290°C and final heating at 290°C for 5 minutes. The flow rate of carrier gas (helium gas) was set to 1.5 ml/minute. A 1 μl of sample was injected in splitless mode, and mass spectra were recorded at 2 scans/sec within a mass- range from 70 to 600.

### LC-MS analysis

The endogenous levels of plant hormones were estimated by LC-MS using a protocol modified from Pan et al. (2010) [35]. Approximately 100 mg of powdered tissue was suspended in 500 μl of extraction solvent (2-propanol/ MilliQ water/concentrated HCL; 2: 1: 0.002 v/v/v), and the suspension was mixed by shaking at 500 rpm for 30 min at 4°C. Thereafter 1 ml of chilled dichloromethane was added to the tube and mixing was continued for another 30 min. The organic and aqueous phases were separated by centrifuging extract at 13000*g* for 5 min at 4°C. Approximately 900 μl of the aqueous phase was transferred to a fresh tube and dried *in vacuo* for 45 min. Dried samples were dissolved in 70 μl of chilled methanol followed by centrifugation at 13,000*g* for 5 min. Thereafter samples were transferred to an injection vial and analysed by using Exactive^™^Plus Orbitrap mass spectrometer (Thermo Fisher, USA) coupled with UPLC (Waters, Milford, MA USA).

The system consisted of Aquity UPLC^™^ System, quaternary pump, and auto-sampler. For separation of hormones, the sample was analysed on a Hyperreal GOLD C_18_ (Thermo Scientific) column (2.1×75 mm, 2.7 μm). A gradient elution program was performed using two solvents system, solvent A- contain ultrapure water with 0.1% formic acid, solvent B- containing acetonitrile with 0.1% formic acid and chromatographic run for 9 min at 20°C. The detection of abscisic acid (ABA), gibberellic acid (GA), jasmonic acid (JA), and salicylic acid (SA) was performed in all ion fragmentation (AIF) mode (range of m/z 50–450) with positive heated electrospray ionization (ESI) in negative ion mode. The zeatin, IAA, indole butyric acid (IBA), methyl jasmonate (MeJA), and epibrassinosteroid were analysed using Turbo Ionspray source in positive ion mode. For both modes following instruments setting was used, capillary temperature—350°C, sheath gas flow (N_2_) 35 (arbitrary units), AUX gas flow rate (N_2_) 10 (arbitrary units), collision gas (N_2_) 4 (arbitrary units) and the capillary voltage 4.5 kV under ultra-high vacuum 4e^-10^ mbar. The levels of endogenous hormones were quantified by using standard curves for the following hormones- ABA, zeatin, IAA, MeJA, and SA.

### Identification of metabolites

The NetCDF files were obtained from ChromaTOF software 4.50.8.0 chromatography version (LECO Corporation, USA) GC-MS were further analysed using MetAlign 3.0 (www.metalign.nl) [36] and MSClust [37] software with a signal to noise ratio of ≥ 2, for baseline correction, noise estimation, alignment and extraction of ionwise mass signal. The mass signals that were present in <3 samples were discarded. The MetAlign results were processed with MSClust software for reduction of data and compound mass extraction. The mass spectra extracted by MSClust were opened in NIST MS Search v 2.2 software for identifying compounds with the NIST (National Institute of Standard and Technology) Library and Gölm Metabolome Database Library (http://gmd.mpimp-golm.mpg.de/). The compound hits which showed maximum matching factor (MF) value (>700) and least deviation from the retention index (RI) was used for metabolite identity. The metabolite levels were quantified by normalizing with the internal standard ribitol. During identification, the metabolites with different trimethylsilyl derivatives were clubbed together as a single entity. In addition, only annotated metabolites were taken for further study. S1 Table lists the detected metabolites with above method of detection with its RI. For assigning the KEGG IDs to the detected metabolites, the publically available platform (http://cts.fiehnlab.ucdavis.edu/) was used.

### Statistical analysis

For statistical analysis, online software MetaboAnalyst 2.0 [38] and Tools for statistical analysis of metabolomics on Microsoft Excel, MS-DIAL [39] was used. To visualize overall clustering of the metabolites, we performed Principal Component Analysis. Heat map and clustered heat map were prepared using publically available Morpheus software (https://software.broadinstitute.org/morpheus.) Hierarchical Clustering was done for rows as well as for columns. K-1 Pearson correlation was used along with dendrograms were cut at 2. For FDR values and Bonferroni correction R language and associated package were used. The PCA graphs were prepared using Microsoft Excel, MS-DIAL [39].

### Metabolic network analysis

To visualize interaction among metabolites and phytohormones, metabolic correlation networks were constructed using Cytoscape software version 3.4.0 (http://www.cytoscape.org/). Interactions of metabolites were studied by comparing cotyledon to callus, and callus to regenerated shoots (RS). Two independent interaction networks were prepared for each of WT, *shr*, and *pct1-2*. For correlation networks, three biological replicates of each stage were taken. Replicates with p value ≤ 0.05 were used to a pairwise correlation using the Pearson's Correlation coefficient (PCC). The associations with PCC ± ≤ 0.9 values were used to create a network. The clusters in the metabolic networks were delineated using Cytoscape plugin, clustermaker 2 version 0.9.5 using default settings. MCODE algorithm was used for making network and clusters. The metabolite and hormone networks between WT and *shr*, and WT and *pct1-2* were similarly plotted by comparing cotyledon, callus, and regenerated shoots of WT with respective mutants (p value ≤ 0.05).

### Dendrogram construction

For dendrogram construction R commands {R version 3.3.1 (2016-06-21)} were used. The igraph package was used to convert edgelists to adjacency matrix, and absolute correlation values were considered. The default method for the dist function was the Euclidean method of distance computation. Dendrapply function was used for coloring the dendrogram. The cutree function was used to cut the tree into several groups by specifying the k = 8 (the cut height).

### Metabolic pathway

The metabolic pathway was manually constructed based on available information from the published literature and network data base e.g. the KEGG (http://www.genome.jp/kegg/) and Solcyc database (http://www.solcyc.solgenomics.net/).

### RNA extraction and transcript analysis

Total RNA was extracted from the cotyledon, callus and regenerated tissue using TRI Reagent (Sigma, USA), according to the manufacturer’s protocol. Contamination of genomic DNA in the isolated RNA was removed by treating with DNAse enzyme (Promega, USA), as per manufacturer’s guideline. cDNA was prepared from 2 μg RNA using Applied Biosystems cDNA synthesis kit. The RT-PCR was conducted using four independent biological replicates with two technical replicates of respective samples at each stage. The ΔCt (fold expression) value of each gene was calculated by normalizing Ct values of each gene to the average expression of two internal control genes (β-actin and ubiquitin3). [ΔCt value = (Ct value of gene)–(average Ct value of control genes)]. The details of RT-PCR protocol are described in Gupta et al. [40]. Primers for *TOR*, *RAPTOR*, *LST8*, *s6K*, *β-actin* and *ubiquitin3* were designed using primer3 software (S2 Table).

## Results

### Callus formation and shoot regeneration

For initiation of callus, ten-day-old cotyledons were used as explants. While *pct1-2* and *shr* mutants differ from wild type (WT) in seedling phenotype, their cotyledons differ from WT primarily in size. Overall, the callus development on CIM was sluggish in mutants, with *pct1-2* being slowest followed by *shr* than WT. Visually, *pct1-2* callus showed less greening than *shr* and WT (S1 Fig). Apart from these, there were no major differences in callus formation amongst WT and mutants. The initiation of shoots from callus on SIM followed nearly similar temporal scale in WT and mutants.

### Alteration of metabolomic homeostasis during callus formation and shoot regeneration

**Fig 1:**
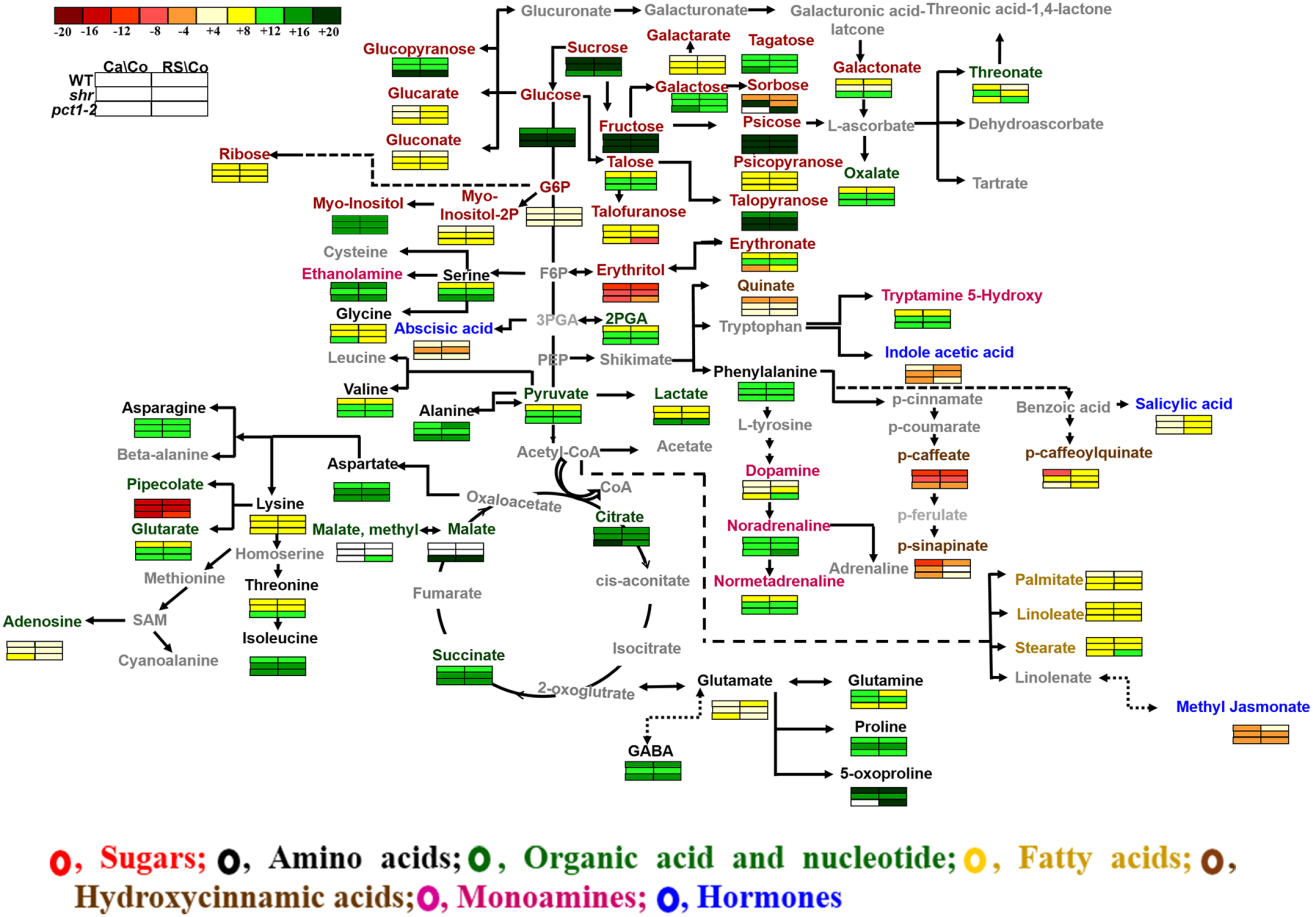
An overview of the metabolic pathways representing relative abundance of metabolites and hormones in callus (Ca) and regenerated tissue (RS) compared to respective cotyledons (Co) in WT, *shr*, and *pct1-2* mutant. The abundance of each metabolite and hormone is represented by a heat map. The grayed metabolites were not detected. The relative changes in the metabolites levels at callus and RS stage were calculated by determining callus/cotyledon or RS/cotyledon ratio of individual metabolites for each genotype. Log2 ratios of fold changes are given by shades of different colours depicted in scale bar (-20 ↔ +20) on top left-hand corner. In the metabolic pathway, sugars (red), amino acids (black), organic acids (green), fatty acids (yellow), monoamines (pink), nucleotides (green), hydroxycinnamic acids (brown) and phytohormones (blue) are shown with respective color. The solid arrow indicates direct conversion between metabolites, the bidirectional arrow shows inter-changeable metabolites, two arrows signify metabolite conversion includes two to three steps and the long dotted arrow indicates conversion includes more than three steps.

Profiling of primary metabolites identified 91 compounds and hormones in cotyledon, callus and regenerated shoot (RS) of WT, *shr*, and *pct1-2* mutants. To provide a broad view, we mapped the identified metabolites and their levels onto general metabolic pathways (Fig 1). An overview of the metabolic pathways revealed that several metabolites are differentially regulated (presented as heat maps) during transformation from cotyledon to callus, and cotyledon to RS in WT and mutants. One of the distinct effects was modulation of glycolysis and TCA cycle with upregulation of metabolites and hormones related to it. The most prominent group of modulated metabolites belonged to carbohydrate metabolism, followed by amino acids and fatty acids. Of interest was the modulation in levels of several metabolites with little-known function in plants such as dopamine, noradrenaline, and normetadrenaline; caffeate and caffeoylquinate; sinapinate, 3-methyl-2-ketopiperazine and pipecolate. The detailed description of differentially regulated metabolites and hormones is provided in later sections.

**Fig 2:**
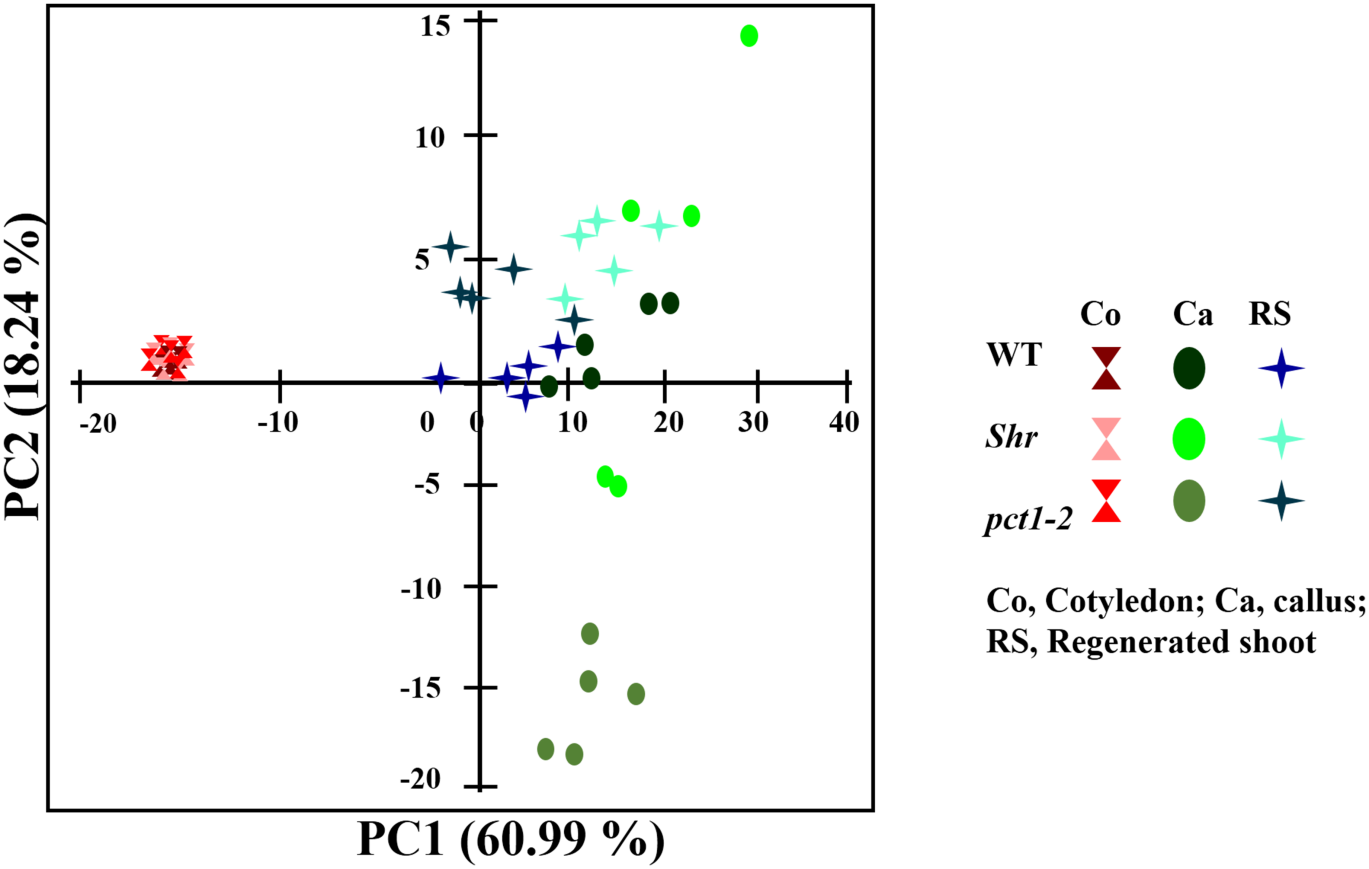
A combined principal component analysis (PCA) of metabolites in cotyledon, callus and RS of WT, *shr*, and *pct1*-2. Each symbol represents one biological replicate. The variance of each component (PC1, PC2) is given within parentheses (%).

To obtain an all-inclusive view of the metabolic changes in callus and RS, principal component analysis (PCA) of the 91 annotated metabolites was carried out. The profiles of cotyledons substantially overlapped indicating that notwithstanding the mutations the metabolite profiles of cotyledons were nearly similar in WT and two mutants. Comparison of PCA profiles further revealed that the first principal component (PC1) resolved tissue/organ specificities between cotyledon, callus, and RS. The metabolites in cotyledons were completely separated from those present in the callus, and the RS was placed between these two. The second principal component (PC2) also resolved similar differences between cotyledon, callus, and RS. The PC2 also indicated that metabolites profiles of RS are closer to each other, whereas the profiles of callus showed wide variation. The profile of *pct1-2* callus distinctly separated from WT with more negative PC2 value, whereas *shr* profile had broader distribution with more positive PC2 values and partially overlapped with WT (S3 Table). The PCA revealed that the metabolites levels in *shr*, *pct1-2* and WT undergo major changes during transformation to callus. During the transition to RS, the metabolite in *shr* and *pct1-2* shifts back to nearly similar profiles. In WT and both mutants, the metabolite shifts follow the above trajectory of changes in an organ-specific fashion (Fig 2)

The differential scores of each metabolite indicated that while most metabolites were causative for PC1, a combination of differentially regulated metabolites contributed to PC2. Loading scores of principal components (S3 Table) reveal that in callus PC2 distinctly resolve the mutants with WT as intermediate. Among the metabolites, pyroglutamic acid, tagatose, fructose, GABA, malic acid, glutamine and D-glucopyranose mainly contributed to the PC2 (S4 Table, Fig 2). In *shr* callus, metabolites such as glucose, alanine, serine, glycine, threonine, proline, glutamic acid, asparagine, 3-methyl 2-ketopiperazine, malate, 1, 3 propandiol display very distinct values compared to *pct1*-2.

### Metabolite profiles of *pct1*-2 and *shr* mutant cotyledons differ from WT

**Fig 3:**
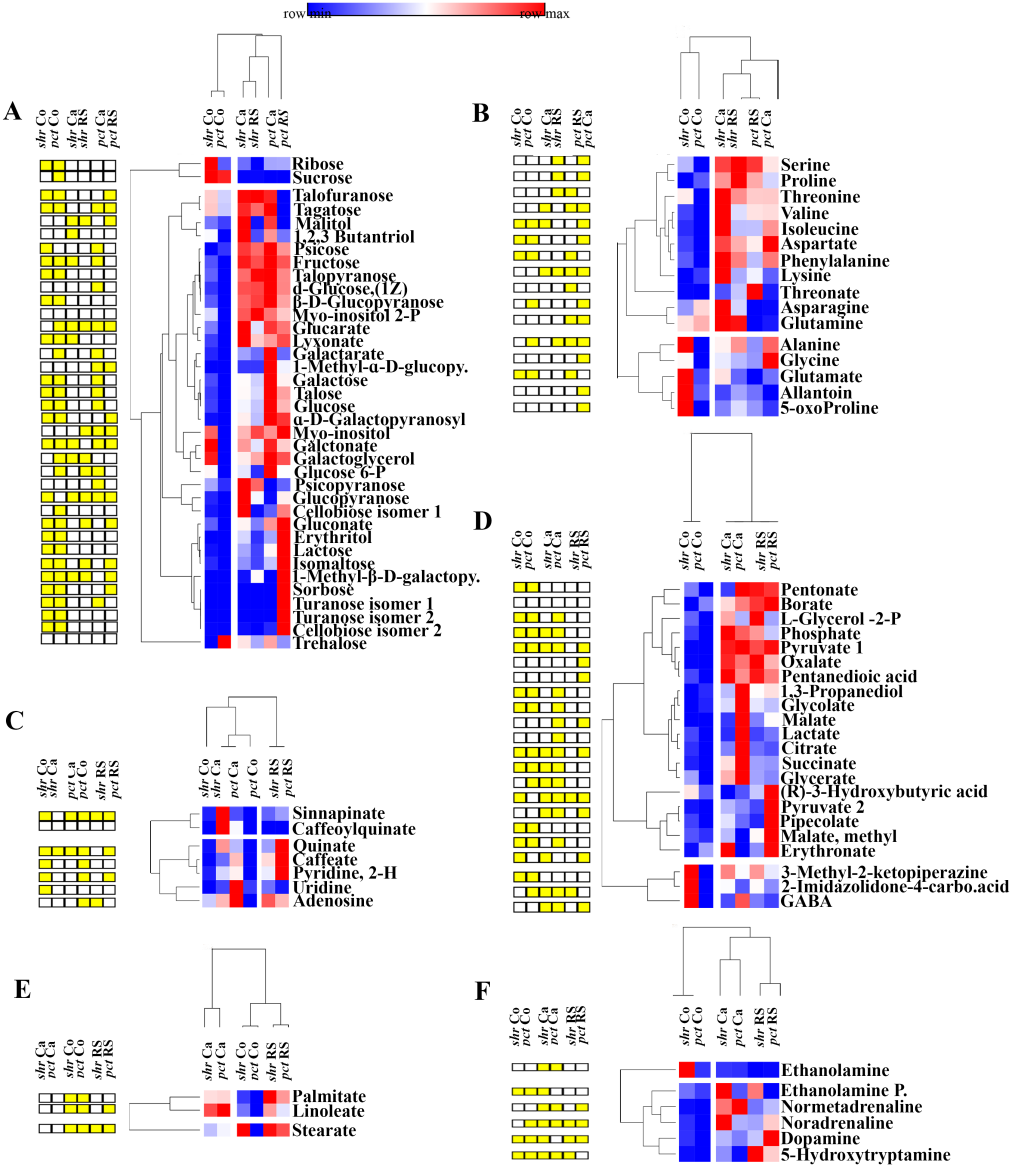
Clustered heat map showing differential expression of metabolites at cotyledon (Co), callus (Ca), and RS stage of *shr* and *pct1*-2 compared to the corresponding stage of WT. **A**-sugars, **B**- amino acids, **C**- nucleotides and hydroxycinnamic acids, **D**- organic acids, **E**- fatty acids and **F**- monoamines. The yellow coloured boxes on left of heat map represent the statistically significant (p ≤0.05) upregulation or downregulation of the respective metabolite. The relative changes between mutant and WT were calculated by determining mutant/WT ratio for individual metabolites at respective stages. Abbreviations: 1-M-ɑ-D-glucopy(1-M-ɑ-D-glucopyranoside); 1-M-β-D-galctopy (1-M-β-D-galctopyranoside); iso(isomer); Ethanolamine P (Ethanolaminephosphate); L-Glycerol 2-P (L-Glycerol 2-Phosphate); 2-Imidazolidone-4-carbo acid (2-Imidazolidone-4-carboxylic acid).

The PCA indicated a shift in profiles of metabolites during the transition to callus and RS. To distinguish the major metabolites contributing to this shift, the relative changes in the metabolite levels were examined by clustering them within their functional classes viz. sugar, amino acids, organic acids, fatty acids, amines and hydroxycinannamic acids. In the cotyledons, while qualitatively metabolites in both mutants and WT were similar, the relative levels of several metabolites in mutants significantly differed from WT. In *pct1-2* and *shr* cotyledons, metabolites like sucrose and glutamine were differentially upregulated. In *pct1*-2 cotyledons levels of trehalose was higher, whereas, in *shr*, ribose, talofuranose, tagatose, myo-inositol, galactonate, galactoglycerol β-D-glucopyranose were higher than WT. Interestingly, the levels of metabolite related to plant immunity like 3-methyl 2-ketopiperazine were higher in *shr* cotyledons. In *shr* cotyledons, levels of amino acids *viz*. glycine, alanine, serine, threonine, glutamine, glutamate, GABA and 5-oxoproline amino acids were higher (Fig 3), whereas, in *pct1-2* cotyledons, only asparagine and glutamine levels were higher than WT (Fig 3). Notwithstanding above, the levels of most metabolites were significantly lower in *pct1-2* (53) and *shr* (37) compared to WT (S5 Table).

### Callus shows up-regulation of carbohydrate metabolism and TCA cycle

**Fig 4:**
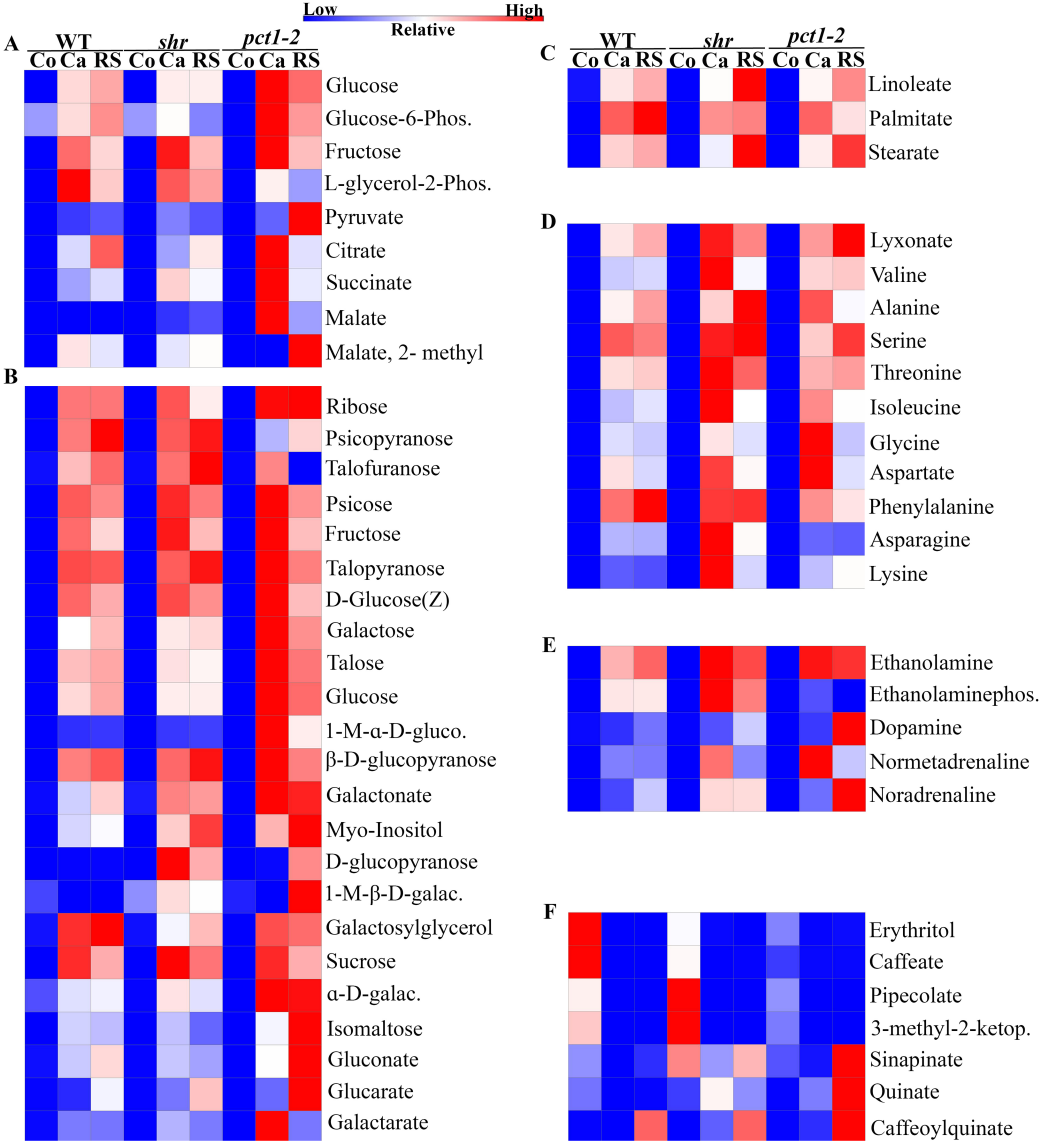
Heat map showing the levels of metabolites (*p* ≤0.05) at cotyledon (Co), callus (Ca) and RS stage of WT, *shr*, and *pct1-2*. The relative levels are indicated by varying color intensity (low-blue, high-red). **A**-Pentose phosphate pathway and TCA cycle, **B**-sugars **C**-fatty acids; **D-** amino acids **E**-monoamines; **F**-cell wall and immunity related metabolites. Abbreviations: 1-M-ɑ-D-gluco (1-M-ɑ-D-glucopyranoside); 1-M-β-D-galac (1-M-β-D-galactopyranoside); ɑ-D-galac (ɑ-D-galactopyranosyl(1,4)-D-galactopyranoside); 3-methyl-2-ketop (3-methyl-2-ketopiperazine); Ethanolaminephos (Ethanolaminephosphate); L-glycerol-2-phos (L-glycerol-2-phosphate).

The induction of callus and RS needs metabolic energy to carry out energetically demanding process of cellular division. Consistent with this, the levels of several intermediates belonging to TCA cycle and Pentose phosphate pathway were up-regulated in callus and RS (Fig 4A). In general, the magnitude of up-regulation of intermediates belonging to these pathways was higher in callus than in RS. Specifically, maximum up-regulation of intermediates was observed in *pct1-2* callus. Interestingly, there was no up-regulation of malate in callus and RS of WT, while it was observed in *shr* and *pct1-2*.

Consistent with the initiation of cellular proliferation, the levels of several carbohydrates involved in cell wall biogenesis were upregulated in callus. Although WT, *shr*, and *pct1*-2 genetically differ, callus formation induced up-regulation of sugars in all three. The levels of 30, 28 and 24 sugars were significantly different in callus and RS of WT, *shr* and *pct1-2* respectively than corresponding cotyledons (S6 Table). Most significant was up-regulation of thirteen different sugars in *pct1-2* callus compared to WT and *shr* callus (Fig 4B). Even in RS, levels of ɑ-D-galactopyranosyl (1,4)-D-galactopyranoside, isomaltose, gluconate and glucarate sugars were up-regulated in *pct1-2* compared to RS of WT and *shr*. In contrast, the levels of sugars in *shr* callus only marginally differed from WT except the high level of D-glucopyranose. In WT callus and RS, the level of 1-methyl ɑ-D-glucopyranoside was marginally upregulated, and that of 1-methyl β-D-galactopyranoside was downregulated compared to both mutants. A notable exception was D-glucopyranose in WT whose levels were not altered both in callus and RS. The alteration in the fatty acids was restricted to only linoleate, palmitate, and stearate, whose levels were upregulated in *pct1-2*, *shr*, and WT callus and RS (Fig 4C).

### Building blocks of proteins were upregulated in callus and regenerated shoot

Similar to carbohydrates, callus formation and shoot regeneration significantly upregulated levels of 11 amino acids in mutants and WT compared to respective cotyledons (Fig 4D; S6 Table). Among these, valine, serine, threonine, isoleucine, phenylalanine, asparagine, and lysine were upregulated in *shr* callus and alanine, glycine and aspartate were upregulated in *pct1-2* callus compared to WT callus. In RS, the overall magnitude of upregulation was lesser than the callus. While alanine, serine, threonine, aspartate, and asparagine were upregulated in *shr*; valine and lysine were upregulated in *pct1-2* compared to WT. The phenylalanine level was higher in RS of WT than in *shr* and *pct1-2*.

### Effect on metabolites related to cell proliferation

The transformation to callus and RS was accompanied by up-regulation of monoamines that act as neurotransmitters in animal system *viz*. dopamine, noradrenaline and normetadrenaline (Fig 4E; S6 Table). The transformation also induced downregulation in levels of specific sets of metabolites related to cell wall lignification (ca. caffeate, sinapinate [except *pct1-2* RS]); related to cell immunity (pipecolate, 3-methyl -2-ketapiperazine); related to cellular proliferation (erythritol, caffeate) in callus and RS of mutants and WT (Fig 4F; S6 Table). The level of quinate was up-regulated in callus and RS of *pct1-2* and *shr* mutant but downregulated in WT. The level of caffeoylquinate was up-regulated in callus and RS of *pct1-2* and *shr* mutant, whereas it was up-regulated in WT callus but downregulated in WT RS.

### Shoot regeneration is associated with upregulation of salicylic acid and zeatin

**Fig 5:**
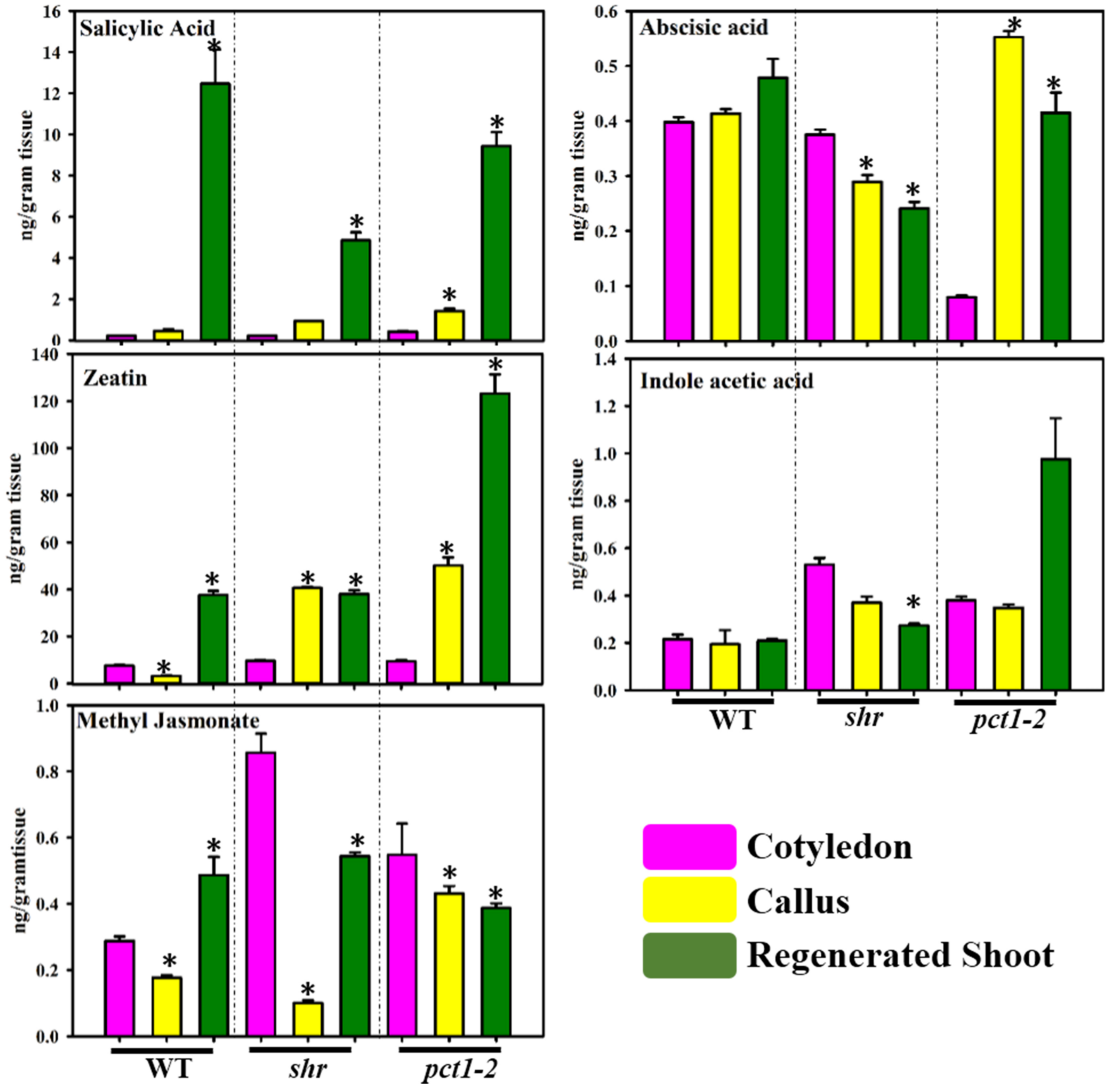
Levels of different plant hormones; salicylic acid (SA), zeatin, abscisic acid (ABA), methyl jasmonate (MeJa) and indoleacetic acid (IAA) in cotyledon (Co), callus (Ca) and regenerated shoots (RS) of WT, *shr* and *pct1*-2 mutant. The asterisk (*) indicates the statistically significant difference in the hormonal level compared to respective cotyledon control (p ≤0.05).

One of the foremost requirements of callus formation and shoot regeneration is the provision of a specific ratio of plant hormones- auxin and cytokinin in the CIM and SIM. In tomato cotyledon, callus, and RS, only five plant hormones viz. IAA, ABA, zeatin, MeJa, and SA were detected, and the levels of other examined hormones- epibrassinolide, IBA, JA, and GA were below the limit of detection. The examination of changes in different hormone levels revealed that there was only a little similarity between WT, *shr* and *pct1-2* except SA levels in callus and RS (Fig 5; S2 Fig). The SA level increased in callus and RS of WT, *shr*, and *pct1-2* with very high upregulation in RS. The ABA level did not show a significant difference in WT, downregulated in *shr* and upregulated in *pct1-2* callus and RS. The zeatin level was upregulated in callus of *pct1-2* and *shr*, and RS of WT, *pct1-2*, and *shr*. The IAA level was upregulated in *pct1-2* RS but downregulated in *shr* RS. The MeJA level was downregulated in callus and RS of *shr* and *pct1-2* and callus of WT but increased in WT RS.

### Comparison of cotyledon and callus indicate major shift in metabolite interactions

To examine the interrelationship between metabolites and hormones during transformation from cotyledon to callus and callus to RS, the correlation networks (r >0.90) were plotted for WT and mutants. The networks included only those metabolites/hormones that showed 1.5 fold higher or lower expressions than the respective controls. Among three cotyledon to callus networks, WT showed highest metabolite connectivity (network density- 0.513; connectivity 6806; nodes 83) followed by *pct1*-2 (network density- 0.343; connectivity 6162; nodes 79) and *shr* (network density- 0.303; connectivity 6162; nodes 79).

**Fig 6:**
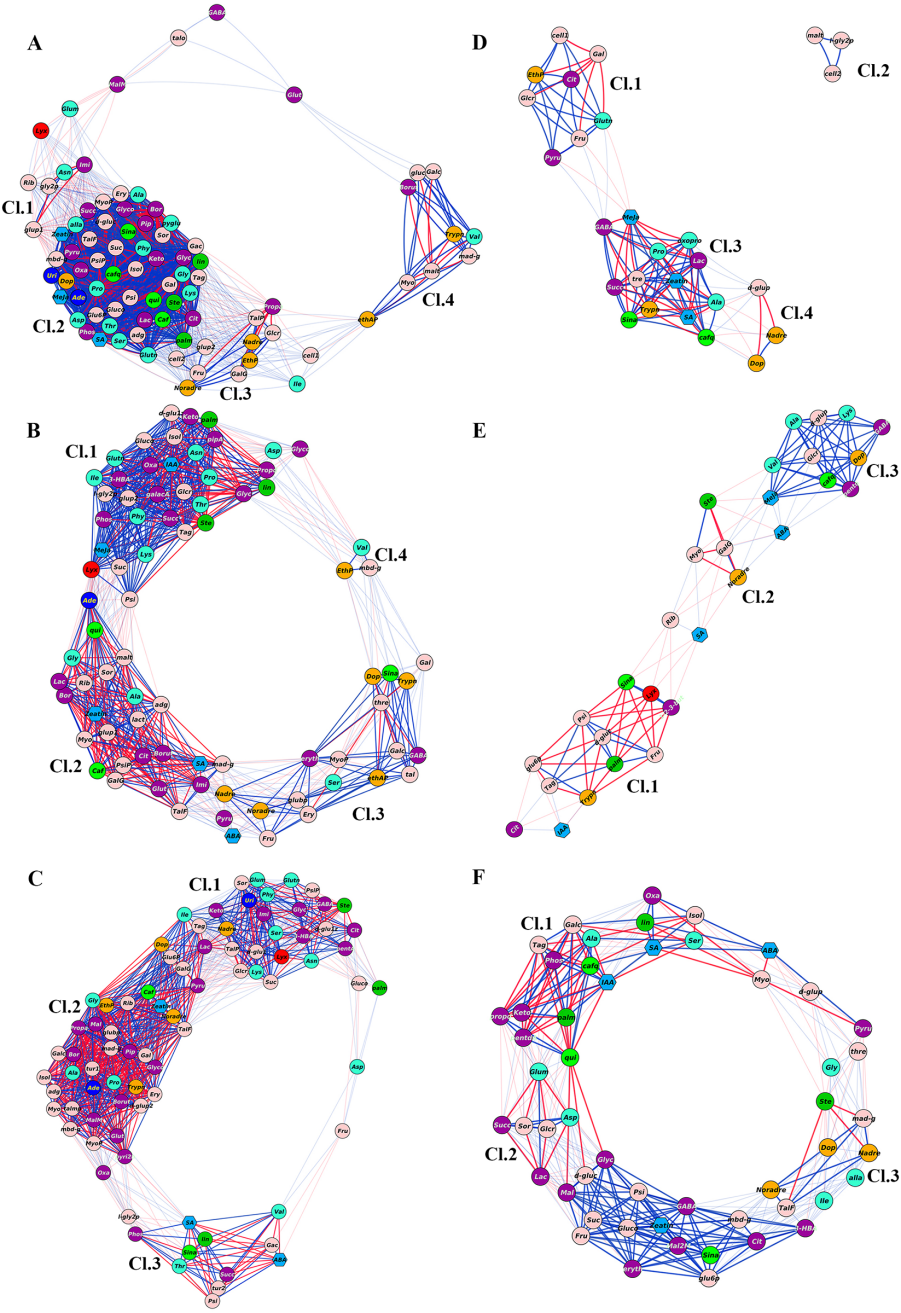
Correlation network analysis of metabolites and hormones in callus/cotyledon and callus/RS of WT (A, D), *shr* (B, E) and *pct1*-2 (C, F) mutant. Networks were plotted using Cytoscape with sugars (light pink), sugar acid (red), amino acids (blue), organic acids (purple), fatty acids (dark green), monoamines (golden yellow), nucleotides (dark blue), hydroxycinnamic acids (grass green) and phytohormones (blue hexagon). Only interactions (p ≤0.05) with r ≥ ±0.9 were used for generating the network. The blue and red lines between the different nodes indicate positive and negative correlations respectively. Independent clusters were connected with each other by faint blue and red lines. The length of the lines indicates the extent of interaction between the connecting nodes. The significant changes in metabolites were calculated by determining callus/cotyledon and callus/RS ratio of individual metabolites for each genotype. (**Cl**- Cluster). The full names of the metabolites depicted on the network are given in supplementary Excel File (S7 Table).

In WT cotyledon to callus networks, four distinct clusters were discernible on the basis of interaction of metabolites (Fig 6A; S3A Fig). The network was populated with more positive (1342) than negative (403) interactions between metabolites. Cluster II with dense interactions consisted of the majority of primary metabolites (48). Interestingly, GABA and glutarate showed only positive interactions. Cluster II also included hormones SA, zeatin, and MeJA and while SA negatively regulated MeJA and zeatin, whereas MeJA and zeatin positively regulated each other. A similar interaction was observed for metabolites where SA regulated metabolites in a fashion opposite to MeJA and zeatin. SA negatively regulated pipecolate, 3-methyl-2-ketopiperazine, caffeate, quinate, sinapinate and caffeoylquinate, whereas MeJA and Zeatin positively regulated these metabolites. The monoamines like dopamine and noradrenaline negatively interacted with SA. The remaining clusters were sparsely populated, and nine metabolites including GABA were not part of any of these clusters (S3A Fig).

Similar to WT, in *shr* cotyledon to callus network too, four clusters were discernible. In addition to SA, MeJa, and zeatin, IAA and ABA also mapped on the *shr* network (Fig 6B; S3B Fig). Consistent with the decrease in network density, in *shr*, the interactions were less dense than WT, which is evident by the massive decline in positive interactions (592) than negative (342) interactions. In addition, the interactions between plant hormones were also altered with MeJa and IAA negatively interacting in cluster I, and SA positively interacting with zeatin in cluster II. ABA positioned (S3B Fig) between cluster II and III negatively interacted with SA. In cluster I, pipecolate and 3-methyl-2-ketopiperazine negatively interacted with IAA. The noradrenaline and normetadrenaline interacted positively and negatively with ABA and SA respectively. Caffeate interacted positively with zeatin and SA, whereas quinate interacted positively with SA and negatively with zeatin.

Unlike WT, in *pct1-2* cotyledon to callus network, three clusters were discernible, but these were populated by different combinations of metabolites (Fig 6C; S3C Fig). Cluster I and II were connected by isoleucine, tagatose, and lactate. Similar to *shr*, the SA and ABA interacted negatively in cluster III but interacted with fewer metabolites compared to *shr*. ABA and SA respectively interacted with sinapinate positively and negatively. On the other hand, zeatin in cluster II negatively interacted with normetadrenaline, noradrenaline, dopamine, caffeate, pipecolate and 3-methyl-2-ketopiperazine.

Compared to cotyledon to callus networks, the callus to RS networks were sparsely populated. However, unlike cotyledon to callus networks, the WT callus to RS network showed least metabolite connectivity than mutants. Among three networks, *pct1*-2 showed highest metabolite connectivity (network density- 0.284; connectivity 2652; nodes 52) followed by *shr* (network density- 0.291; connectivity 812; nodes 29) and WT (network density- 0.357; connectivity 512; nodes 26).

Similar to cotyledon to callus network, in WT callus to RS networks too, SA, zeatin, and MeJA mapped in the same cluster (Cluster III) (Fig 6D, S3A Fig). However, zeatin negatively regulated MeJA and SA whereas SA and MeJa positively regulated each other. SA positively regulated dopamine, noradrenaline and negatively interacted with caffeoylquinate and sinapinate. Zeatin positively regulated sinapinate and caffeoylquinate. MeJa negatively regulated sinapinate and GABA. The remaining clusters were sparsely populated and lacked any phytohormones (S3A Fig)

In callus to RS network of *shr* ABA, MeJa, SA and IAA mapped on the network (Fig 6E, S3B Fig), but these showed no self-interaction, except positive interaction between MeJa and ABA. Except MeJa other phytohormones were not present in the main clusters. The interaction of IAA was with citrate, palmitate, tagatose, glucose-6-phosphate and 5-hydrooxytryptamine. SA negatively interacted with sinapinate, normetadrenaline, whereas ABA and MeJa interacted positively with normetadrenaline, caffeoylquinate, and dopamine.

The callus to RS network of *pct1-2* was more densely populated with ABA, IAA, zeatin and SA mapping on the network (Fig 6F, S3C Fig). In cluster I SA interacted positively with IAA and ABA whereas zeatin did not show any interaction with above hormones. Zeatin positively interacted with sinapinate, normetadrenaline and GABA. SA negatively regulated caffeoylquinate. IAA positively regulated 3-methyl-2-ketopiperazine and negatively regulated quinate and caffeoylquinate. To decipher the interrelationship among the metabolites, a hierarchical dendrogram was constructed for WT (S4A Fig), *shr* (S4B Fig) and *pct1*-2 (S4C Fig). The metabolites and hormones in dendrogram interacted with each other in a fashion that was a close replica of the above metabolic networks.

To ascertain metabolic shifts specific to *shr* and *pct1-2* mutants, we also plotted networks with metabolite correlations present only in the respective mutant (S5 Fig). Interestingly, the more interactions were observed in the cotyledons of mutants (*shr* +222, -02; *pct1-2* +221, -23) (S5A and S5D Fig), whereas in callus (*shr* +30, -02; *pct1-2* +21, -47) (S5B and S5E Fig), and RS (*shr* +27, -04; *pct1-2* +71, -09) (S5C and S5F Fig) fewer interactions were present. Surprisingly, both *shr* and *pct1-2* mutants showed preponderance of positive interactions at all stages barring callus of *pct1-2* mutant. Importantly only few interactions were observed for plant hormones including IAA.

### Callus formation and shoot regeneration modulate *LST8* and *TOR* transcript- constituent of TOR signalling pathway

**Fig 7:**
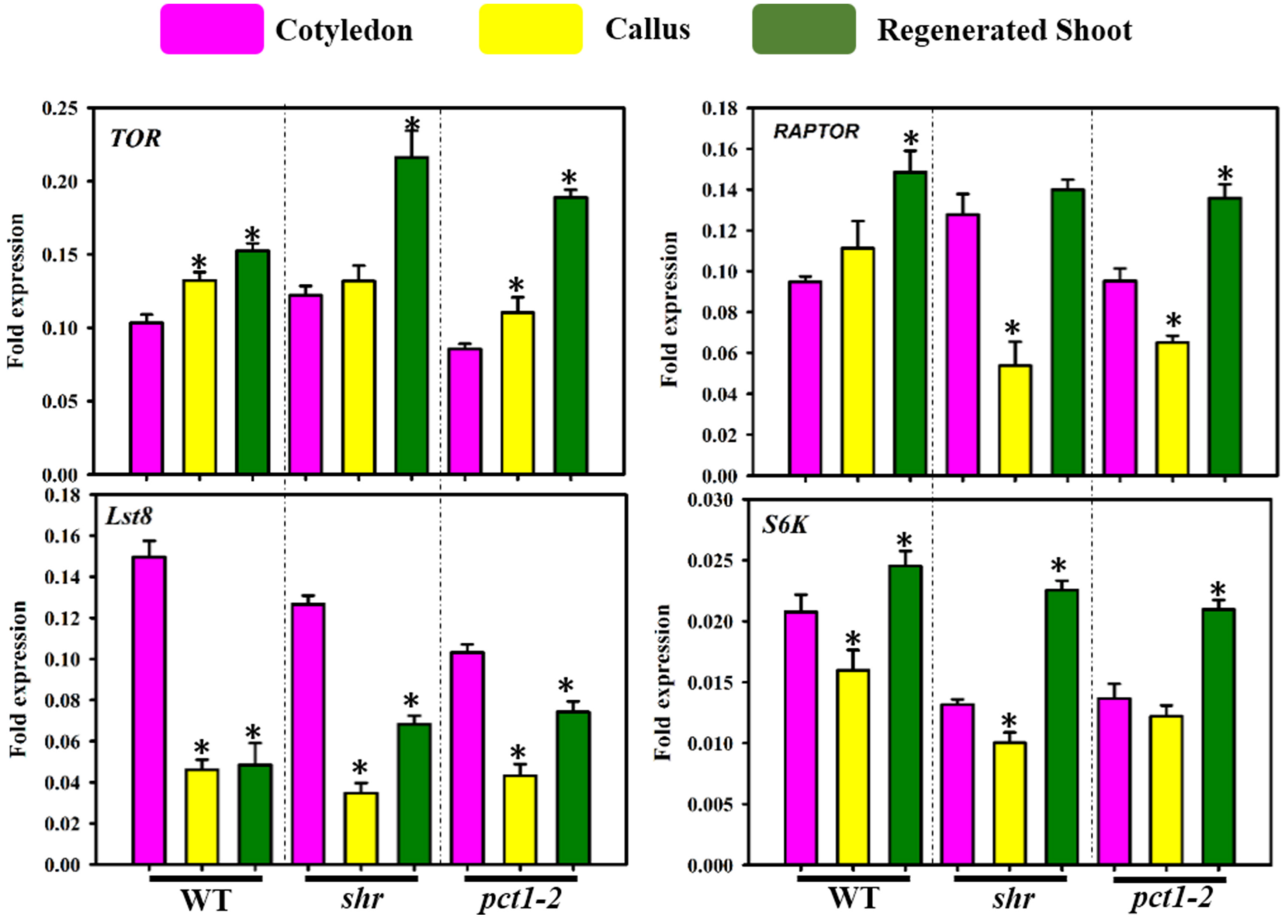
Relative transcript levels (Δt) of *TOR*, *RAPTOR*, *LST8* and *S6K* genes of the TOR-signalling pathway in cotyledon (Co), callus (Ca), and regenerated shoots (RS) of WT, *shr*, and *pct1-2* mutants. The asterisk (*) indicates the statistically significant difference in the transcript level compared to respective cotyledon control (p ≤0.05).

In plants, TOR signalling pathway has been implicated in the regulation of several metabolic pathways [41]. We compared transcript levels of four genes- *TOR*, *RAPTOR*, *LST8*, and *S6K* reportedly associated with TOR signalling in plants in cotyledon, callus, and RS (Fig 7; S6 Fig). The genes were identified based on homology searches with other plant species. Among these genes, *LST8* level drastically declined during callus formation in WT and also in mutants, followed by moderate upregulation at RS. In contrast, the *TOR* level increased in RS of WT and mutants. However, *TOR* showed only a moderate increase in callus of WT and mutants. The *RAPTOR* level specifically declined in *pct1-2* and *shr* callus followed by an increase at RS. The level of *S6K* declined at callus stage and thereafter upregulated at RS.

## Discussion

Plant cells are totipotent and plants can be regenerated from a variety of tissues on culture media supplemented with appropriate hormones. Current evidences suggest that transdifferentiation of mature tissues to callus involves a molecular reprogramming. The effect of this reprograming on the metabolite levels/pathways and endogenous hormone levels remains largely unexplored. Here, we examined correlations between metabolites and hormone levels in WT to decipher how metabolomic homeostasis differs in the callus and the regenerated shoot in WT. We also compared the shifts in metabolic homoeostasis in WT with *pct1-2* and *shr* mutants to decipher how the metabolic homoeostasis is affected by enhanced transport of auxin and over-accumulation of NO in these mutants.

### Callus shows a major shift in metabolite profiles

Both *shr* and *pct1-2* mutants show sluggish growth and pleiotropically affect plant development which is likely related to enhanced auxin transport in *pct1-2* [26], and elevated NO levels in *shr* mutant [31]. Consistent with their slow growth, initiation of callus was also slower in mutants. The PCA profiles of callus and RS of *shr*, *pct1-2*, and WT were distinctly different from cotyledons indicating the transition to these stages causes a major shift in metabolite profiles of mutants and WT. Nevertheless, the tissue-specific characteristics are still retained in the metabolite profiles, as evident by PCA profiles of cotyledons, callus, and RS of WT and mutants. The PCA profile of each stage was more alike in WT and mutants, whereas profiles of cotyledons, callus and RS distinctly differ from each other. Above shifts in PCA profile are in consonance with the difference in metabolism between differentiated cells of cotyledon and actively dividing cells of callus and RS.

### Transformation to callus involves upregulation of anabolism

The transformation of cotyledon to callus, and later to RS involves two opposite sets of cellular programs [42]. The proliferating cells in callus apparently have genetic identity similar to primordial cells of emerging lateral roots [7], whereas organogenesis in RS is associated with localized expression of a subset of *PLETHORA* genes [16]. In either case, cellular programing requires a shift in metabolic networks to provide the energy needed for cell division/expansion and differentiation. In the animal system, this is regulated by reprogramming of anabolism by the action of mTOR1 that acts as the master regulator of this shift [43]. In plants, evidences have been emerging that target of rapamycin (TOR) kinase reprograms transcriptional networks regulating metabolism directed for energy and cellular mass production for developmental transitions [23]. Consistent with this role, increases in TOR expression stimulates root and shoot growth [24].

Consistent with the need for metabolic rewiring for sustaining the growth, the levels of several carbohydrates and TCA cycle intermediates were upregulated in callus and RS to provide the energy needed to sustain the growth. In plant cells, an extensive amount of carbohydrates is also required for biosynthesis of cell walls, a pathway that is regulated by TOR signalling [44]. In conformity with this need, many of the upregulated carbohydrates in the callus and RS likely provide precursors for the cell wall biosynthesis.

To meet the demand of intensive protein synthesis, the levels of several amino acids were upregulated in the callus and RS. The high levels of glutamate and GABA indicate an active operation of C/N shunt in the callus cells. Emerging evidences indicate that GABA inhibits root growth via modulation of anion channels [45], and high concentration of GABA inhibits root elongation [46]. Since callus cells are physiologically closer to lateral root primordial [7], the GABA upregulation may be part of metabolomic homeostasis to promote root cell specific identity in the callus.

The callus formation also reduces several metabolites derived from the hydroxycinnamic acid (caffeate and caffeoylquinate) pathway that have a role in cell wall lignification [47], [48]. The mainframe of cell wall- cellulose in combination with other metabolites and proteins undergoes several modifications such as extension and lignification. The walls of callus cells are generally less rigid than the organs as evident by characteristics of callus being a friable, soft mass of unorganized tissue [9]. The reduction in levels of these precursors for lignification is consistent with above property of callus cell walls. The need for cell wall lignification reappears in RS where levels of caffeoylquinate and sinapinate, which are involved in rigidification of cell walls, increase to promote tissue differentiation associated with organogenesis.

### Callusing lowers the metabolites levels related to cellular immunity

Apparently, callus formation was accompanied with lowering of cellular immunity as evident by reduction in the level of pipecolate which reportedly acts in defence amplification and priming of systemic acquired resistance in plants [49], [50]. Considering that pipecolate seemingly inhibits cell division [51], it is possible that reduction in its abundance may also be related to ongoing active cell proliferation in callus and RS. Similar to pipecolate, the decline in levels of 3-methyl-2-ketopiperazine may be related to lowering of immunity, as evidences are emerging that diketopiperazine and its derivative have antimicrobial role in a wide range of plant species (European Patent EP2594134A2). In plants, emerging evidences support that growth is inversely correlated with the immunity and the trade-off between these two responses is achieved by integrating hormonal/biotic signalling with developmental programs [52], [53]. The reduction in JA level and no significant change in SA level (except *pct1-2*) in callus, and immunity-related metabolites are consistent with the notion that the proliferating cells preferably allocate resources for growth than the defence responses.

### Upregulation in monoamines levels may have a regulatory function

Higher accumulation of monoamines (dopamine, noradrenaline, normetadrenaline) in callus and later in RS may be of physiological significance. Their accumulation is seemingly linked with ongoing cell division in callus and cell division/differentiation in RS. Apparently, these molecules may play a substantive role in above process either directly or in coordination with plant growth hormones. Such an interaction of dopamine, adrenaline, and noradrenaline with plant hormones was suggested by Protacio et al. (1992) [54], as these monoamines promoted the growth of tobacco thin layer cell culture (TCLs) and *Acmella oppositifolia* hairy root cultures only in the presence of IAA and kinetin. It was suggested that inclusion of dopamine in media may have regulated the endogenous level of IAA probably by inhibiting IAA oxidase activity. These monoamines may also have alternate functions, as hydroxycinnamoyl amides of dopamine and noradrenaline in tomato seemingly also have antibacterial and antioxidative roles [55], [56].

### Transformation affects the levels of endogenous hormones

Though the relative ratio of exogenous auxin and cytokinin plays a key role in callus formation and organogenesis, little is known about endogenous levels of hormones during this process. It is suggested that above exogenous hormonal ratio regulates biosynthesis and distribution of endogenous hormones leading to initiation and sustenance of callus [14]. In tomato cotyledon/callus/RS, out of nine hormones examined, only IAA, ABA, zeatin, MeJa, and SA were detected. So far in callus, auxin and cytokinin levels have been indirectly inferred by visualizing auxin responsive (*DR5*::*GFP*) or kinetin responsive (*ARR5*::*GFP*) gene expression [14]. Notwithstanding presence of IAA in CIM, the IAA levels in WT and mutants callus were nearly similar to respective cotyledons. The initiation of shoot primordia involves YUCCA regulated auxin biosynthesis and transport facilitated by PIN1 transporter [57]. In conformity with above, IAA levels were higher in RS of *pct1-2* mutant that over-accumulates PIN1 protein [27]. The reduction in IAA levels in RS of *shr* mutant is likely related to the multifaceted interactions of NO with plant hormones [31]. In contrast to auxin, the kinetin responsive gene expression is strongly distributed throughout the callus and during organogenesis, it is localised to the shoot progenitor cells facilitating cell division [14]. The patterns of zeatin accumulation are broadly in conformity with above localization, where barring WT callus, its levels were upregulated in callus of mutants, and RS of mutants and WT.

Though there is no *a priori* information about the role of endogenous ABA, SA, JA and MeJa in the process of callus formation and RS, it may be surmised that these may act independently or in combination with other hormone/metabolites influence the above process. In *shr*, *pct1-2* and WT, the ABA level did not follow a consistent pattern; nevertheless, it is reported that exogenous ABA alone [58] or together with MeJA [59] acts as a negative regulator of callusing. In contrast, the SA levels were distinctly upregulated in RS, while those of MeJA declined. In tobacco TCL, exogenous MeJa disrupts shoot formation by over-inducing mitotic activity and cell expansion [60]. The lowering of MeJa level in callus and RS thus may be related to the reduction in mitosis and cell expansion to induce organogenesis.

### Correlation networks reveal robust regulation of metabolomic homeostasis

Considering that callus cells are more akin to root cells [7], hormone regulation of cell division in the callus is likely to be similar to root cells. Several evidences indicate that in Arabidopsis, cell division in root meristem is modulated by a complex interaction between ABA, cytokinin, brassinosteroids, IAA and GA [61] and this regulation varies along the differentiation zones of root cells [62]. Likewise, the organogenesis on shoot apical meristem also involves a complex interaction between endogenous hormones and the metabolites during callus formation and RS. Metabolites, as the final product of gene(s) expression, build the cellular structures and thus their connection with hormones is important to identify the key participants in this process. Considering this, we examined the correlation based networks (CN) between hormones and metabolites to decipher the regulatory mechanisms underlying callus formation and RS.

The transformation from cotyledon to callus revealed a major shift in metabolic network profiles with the network of WT being denser than *shr* and *pct1-2*. The observed shift is consistent with predominant upregulation of anabolism to sustain the growth and cell division in the callus. The difference in density of networks between WT and mutants likely reflects the need to balance the metabolomic homeostasis that is probably affected by *shr* and *pct1-2* mutations. The above balancing is presumably achieved by altering the hormonal interactions with the metabolites. Such hormonal alteration is overtly evident as WT, *shr*, and *pct1-2* callus networks share only zeatin and SA as common hormones. While WT and *shr* share MeJa, *pct1-2* and *shr* share ABA, and IAA exclusively maps only in *shr*. It can be surmised that hormonal shift is part of compensation mechanism to overcome the likely adverse effect of *shr* and *pct1-2* mutations. Such a compensation mechanism is essential for living cells to build metabolic networks strongly robust against mutations to sustain essential functions and cellular metabolism [63]. The observed compensation may stem from genetic redundancy, where a mutation has little effect on the overall development of organism due to compensation by other genes [64]. The existence of very few unique interactions in networks of cotyledons, callus and RS of WT with *shr* and *pct1-2* mutants is also in conformity with the robust regulation of cellular homeostasis.

In eukaryotes, the TOR signalling pathway is considered to play a critical role in regulating cellular metabolism [41], [65]. Down-regulation of the *TOR* gene triggered massive shifts in the primary metabolites with increase in the levels of several amino acids, TCA cycle intermediates and GABA in Arabidopsis [66]. Likewise, increase in amino acids, and other metabolites were also observed in long day-grown plants of *lst8* mutants of Arabidopsis [67]. Tomato callus too shows a similar metabolic shift with increase in levels of amino acids, GABA, and TCA cycle intermediates. Therefore, it is logical to assume that above metabolic shift in callus may be related to alteration in the TOR signalling pathway. Consistent with this view, callus formation is associated with a severe decline in expression of *LST8* transcript in WT and both mutants. It is believed that TOR associates with the RAPTOR and LST8 to form a functional TORC1 complex [23]. Thus, the reduction in the level of *LST8* may, in turn, modulate TOR signalling as LST8 is required for optimal functioning of TORC1 complex and associated signalling. A link between *TOR* silencing and calli formation has been observed in Arabidopsis seedlings where prolonged ethanol-induced silencing of *TOR* initiates calli like structure on hypocotyl and near apical meristem [68]. In addition, it is reported that TOR and S6K are involved in a pathway to repress cell proliferation [22]. Consistent with this, observed lowering of *S6K* transcript levels during callus formation may be linked with the sustenance of cell proliferation in the callus.

Current evidences indicate that TOR signalling pathway in association with hormonal regulation and cell proliferation control pathways combinedly balance resources for growth and proliferation of cells [22]. The increase in the number of hormonal regulators in the network and the shift in their interaction with metabolites in callus network in *shr* and *pct1-2* mutant is in consonance with the need for alternate routes to sustain the metabolomic homoeostasis. In yeast, several loss-of-function mutations have a smaller effect on the growth and development as the metabolic network is sustained by alternate routes [69], [70]. In callus network, the robustness is likely attained by remodelling of hormonal and metabolite interactions in mutants by a shift in positive and negative interactions and usage of alternate routes to sustain the metabolomic metabolism. Considering that TOR acts as a central regulator of metabolic networks, this remodelling may involve hormonal regulation of TOR signalling pathway. Evidences indicate that auxin specifically activates TOR/S6K1 signalling by regulating phosphorylation of S6K1 and promoting association of TOR with polysomes to stimulate mRNA translation [71]. Likewise, nitric oxide may regulate TOR signalling pathway by nitrosylation of proteins and interaction with other plant hormones [72], [28]. Such modulation is apparent in *shr* callus where IAA also maps on network presumably to counteract the effects of elevated NO on the cellular homeostasis.

In contrast to callus, the transformation from callus to RS elicited only a moderate change in metabolic networks as evident by networks with very few nodes. Among these, *pct1-2* had most dense network and WT had most scattered network. Ostensibly, callus to RS transformation involved continuation of the cell divisions, which was already initiated during callus formation and only additional change was initiation of organogenesis and shoot growth. In RS, unlike callus, no single hormone was common between WT, *shr*, and *pct1-2* networks. Moreover, the interactions among hormones were completely different than callus and also between WT and mutants. It can be surmised that similar to the callus, in RS too, the difference between WT and mutant metabolic networks likely results from compensation to overcome the effect of *shr* and *pct1-2* mutations.

### Conclusions

In summary, our study indicates that in the callus, and in regenerated shoot, other than zeatin and auxin that are present in culture media, the endogenous hormones also play an important role in maintaining metabolomic homeostasis. The robustness of the metabolism and cellular proliferation is maintained by remodelling of hormonal interactions with metabolites. This remodelling likely overcomes the effect of the excess nitric oxide in *shr* and enhanced auxin transport in *pct1-2* mutants on metabolomic homeostasis by a compensation mechanism which remains to be deciphered, but may involve regulation of TOR signalling. Our results also indicate that proliferation of cells in the callus also involves the reduction in the cell wall lignification and lowering of the cellular immunity. It has to be noted that by no means our analysis is comprehensive, as our study includes only a limited number of metabolites/hormones and their interactions. Our analyses do not include the metabolites/ hormones present in very low abundance or those that could be detected by alternate methods. A more detailed analysis at systems level integrating transcriptome, proteome and metabolome may provide more information about how metabolomic homeostasis is regulated during callus formation and shoot regeneration.

## Supporting information

S1 Table List of metabolites, methods of identification and categories of the metabolites detected in the study. SD, metabolites detected based on standards.
(XLSX)

S2 Table Sequences of primers used for the quantitative real-time PCR of TOR signaling pathway genes. (PDF)

S3 Table List of samples and its score for PC1 to PC5 used for combined principal component analysis. (PDF)

S4 Table List of metabolites and PC1 and PC2 loading score for principal component analysis for aggregated analysis. (PDF)

S5 Table List of metabolites and their relative levels in cotyledon, callus and RS of *shr* and *pct1*-2 in comparison to WT tissue/organ of respective stage. (PDF)

S6 Table List of metabolites and phytohormones, their levels in five replicates of cotyledon, callus, and in RS of WT (A), *shr* (B) and *pct1-2* (C). (XLSX)

S7 Table Full names of metabolites along with the abbreviated names depicted on the networks. (XLSX)

S8 Table List of metabolites with p-value, FDR (q) value along with the Bonferroni correction. (XLSX)

S1 Fig Different stages of culture of tomato WT, *shr* and *pct1-2* mutants. **(A)** Phenotype of 9–10 days old cotyledons used as explants. **(B)** Trimmed cotyledons placed on CIM **(C)** Initiation of callus after 20–25 days of incubation on cotyledon margin **(D)** Callus after second subculture on SIM **(E)** Differentiated tissue.
(TIF)

S2 Fig Heat map showing the levels of hormones (*p* ≤0.05) at cotyledon (Co), callus (Ca) and RS stage of WT, *shr*, and *pct1-2*. The relative levels are indicated by varying color intensity (low-blue, high-red) with reference to respective cotyledons. The coloured hexagons on right of heat map represent the statistically significant (*p* ≤0.05) upregulation (yellow) or downregulation (red) of respective hormones.
(TIF)

S3 Fig Correlation network analysis of metabolites and hormones in callus/cotyledon and RS/callus of WT (A), *shr* (B) and *pct1-2* (C) respectively. Networks were divided into independent clusters using Cytoscape. Metabolites not mapping in any cluster are present as independent entities. Networks were plotted using Cytoscape with sugars (light pink), amino acids (sea green), organic acids (purple), sugar acid (red) fatty acids (dark/lime green), monoamines (dark golden), nucleotides (dark blue), hydroxycinnamic acids (lawn green) and phytohormones (blue hexagon). Only interactions (*p* ≤0.05) with r ≥ ±0.9 were used for generating the network. The blue and red lines between the different nodes indicate positive and negative correlations respectively.
(PDF)

S4 Fig Hierarchical clustering of metabolites. The clustering significantly changes during transition from cotyledon to callus and callus to regenerated shoots of WT (**A**), *shr* (**B**) and *pct1-2* (**C**) respectively. Metabolites and hormones grouping in independent clusters were colored with different colors. In each figure the color code of cluster is mentioned.
(PDF)

S5 Fig Correlation network analysis of metabolites and hormones in cotyledon of *shr*/WT (Fig A) and *pct1-2*/WT (Fig, D); callus of *shr*/WT (Fig B) and *pct1-2*/WT (Fig, E); and regenerated shoot of *shr*/WT (Fig C) and *pct1-2*/WT (Fig, F). Networks were plotted using Cytoscape with sugars (light pink), amino acids (blue), organic acids (purple), fatty acids (dark green), monoamines (golden yellow), nucleotides (dark blue), hydroxycinnamic acids (grass green) and phytohormones (blue hexagon). Only interactions (p ≤0.05) with r ≥ ±0.9 were used for generating the network. The blue and red lines between the different nodes indicate positive and negative correlations respectively. The significant changes in metabolites and hormones were calculated by determining *shr*/WT and *pct1*-2/WT ratio of individual metabolites for each stage. The full names of the metabolites depicted on the network are given in supplementary Excel File (S7 Table).
(TIF)

S6 Fig Heat map showing the genes expression of TOR signaling pathway (*p* ≤0.05) at cotyledon (Co), callus (Ca) and RS stage of WT, *shr*, and *pct1-2*. The relative levels are indicated by varying color intensity (low-blue, high-red) with reference to respective cotyledons. The coloured hexagons on right of heat map represent the statistically significant (*p* ≤0.05) upregulation (yellow) or downregulation (red) of respective genes.
(TIF)
