# Supplementary material for: Metabolomic homeostasis shifts after callus formation and shoot regeneration in tomato
Source: PLoS One. 2017 May 8;12(5):e0176978. doi: 10.1371/journal.pone.0176978 (PMC5421760; doi:10.1371/journal.pone.0176978)
Supplement: S2 Table — (PDF) [file pone.0176978.s002.pdf]

S2 Table: Sequences of primers used for quantitative real-time PCR of TOR signaling pathway genes.

**Name of Primer/Gene ID**	**Orientation**	**Sequence of primer(5′→3′)**
TOR/Solyc01g106770.2	Forward	CCAACTTGTCTCAAAGGAGCTTA
	Reverse	TTGTGTTCACCAAAATACAGACG
RAPTOR/Solyc09g014780.2	Forward	GCCAAATCTGTTAAGTCAACTGG
	Reverse	GTCCTCCATTCATATCAAACCAA
Lst8/Solyc03g059310.2	Forward	AGTCAAGATTTGGAATGTGGATG
	Reverse	TCAGAAGATGCTGTGATGAGAAA
s6K/Solyc03g095510.2.1	Forward	GCATAATGCACAGGGATCTAAAG
	Reverse	CAAAGAGTTGGATCTCGTGTTCT
β-ACTIN/FJ532351.1	Forward	GTCCCTATTTACGAGGGTTATGC
	Reverse	CAGTTAAATCACGACCAGCAAGATT
UBIQUITIN 3/X58253.1	Forward	GCCGACTACAACATCCAGAAGG
	Reverse	TGCAACACAGCGAGCTTAACC
