# Supplementary material for: Metabolomic homeostasis shifts after callus formation and shoot regeneration in tomato
Source: PLoS One. 2017 May 8;12(5):e0176978. doi: 10.1371/journal.pone.0176978 (PMC5421760; doi:10.1371/journal.pone.0176978)
Supplement: S3 Table — (PDF) [file pone.0176978.s003.pdf]

S3 Table: List of samples and its score for PC1 to PC5 used for preparation of PCA graph (Fig 2). For each samples five replicates, designated as 1, 2... 5 were used. Values were obtained using statistical analysis software (MS-DIAL) (37).

S.No.	Identity of Sample	t1 (PC1)	t2(PC2)	t3(PC3)	t4(PC4)	t5(PC5)
1.	WT cotyledon 1	-14.9955	0.865342	-1.21935	-0.40824	-0.91243
2.	WT cotyledon 2	-14.9926	0.880337	-1.22462	-0.42216	-0.90282
3.	WT cotyledon 3	-14.9962	0.875863	-1.2245	-0.4156	-0.90204
4.	WT cotyledon 4	-14.9952	0.878954	-1.22667	-0.42075	-0.90413
5.	WT cotyledon 5	-14.9946	0.876001	-1.22758	-0.41914	-0.9046
6.	WT callus 1	14.65433	2.265387	-8.96241	0.980778	2.012335
7.	WT callus 2	6.953675	1.341199	-6.66887	1.25539	2.214846
8.	WT callus 3	13.75756	2.188601	-8.8133	2.241374	1.606939
9.	WT callus 4	5.073209	-0.58268	-5.20761	-0.49456	2.697948
10.	WT callus 5	8.015938	-0.18716	-6.25933	0.284866	2.123866
11.	WT regenerated shoot 1	-2.92366	0.863427	-3.47241	-0.18259	1.759262
12.	WT regenerated shoot 2	6.121923	0.803847	-5.25238	-0.0917	3.245501
13.	WT regenerated shoot 3	3.52532	-0.60289	-3.79546	-0.59502	3.19924
14.	WT regenerated shoot 4	3.107793	0.107602	-4.35491	-0.38583	2.520723
15.	WT regenerated shoot 5	3.460388	0.649344	-4.51336	-0.16466	2.879525
16.	*shr* cotyledon 1	-14.9995	0.883323	-1.22817	-0.42742	-0.91838
17.	*shr* cotyledon 2	-15.0027	0.884768	-1.22778	-0.42871	-0.92747
18.	*shr* cotyledon 3	-15.0076	0.884874	-1.22868	-0.42579	-0.93481
19.	*shr* cotyledon 4	-15.0068	0.882585	-1.22815	-0.42565	-0.93152
20.	*shr* cotyledon 5	-15.0048	0.881963	-1.22731	-0.42515	-0.92922
21.	*shr* callus 1	15.62906	5.216974	4.714867	-5.13575	-1.59706
22.	*shr* callus 2	11.76894	6.049706	2.508966	-2.75255	-1.92895
23.	*shr* callus 3	6.19495	-2.95889	8.673294	-11.0439	1.973481
24.	*shr* callus 4	30.56472	17.28262	-4.37012	0.646705	-4.9618
25.	*shr* callus 5	6.152444	-3.31047	9.814799	-10.4184	2.167158
26.	*shr* regenerated shoot 1	6.047735	5.328792	1.801241	-1.72482	-0.80156
27.	*shr* regenerated shoot 2	6.446259	2.748685	3.609126	-1.09924	-0.97289
28.	*shr* regenerated shoot 3	4.781036	4.723015	1.631016	-1.91518	0.455321
29.	*shr* regenerated shoot 4	12.99782	5.558468	-0.56217	-1.12934	-0.23472
30.	*shr* regenerated shoot 5	10.23333	3.308742	3.397117	-0.31282	-2.33282
31.	*pct* cotyledon 1	-14.9734	0.873323	-1.2347	-0.41776	-0.85124
32.	*pct* cotyledon 2	-14.9727	0.874104	-1.23578	-0.41926	-0.85041
33.	*pct* cotyledon 3	-14.9707	0.8744	-1.23127	-0.41834	-0.84907
34.	*pct* cotyledon 4	-14.9719	0.869432	-1.23315	-0.41664	-0.85545
35.	*pct* cotyledon 5	-14.9742	0.874245	-1.23572	-0.41923	-0.853
36.	*pct* callus 1	9.825215	-11.6716	-2.69388	1.95548	-1.95057
37.	*pct* callus 2	4.944697	-18.3946	2.194846	-1.45598	0.128084
38.	*pct* callus 3	6.274541	-19.3407	2.663726	-1.36558	1.579887
39.	*pct* callus 4	14.71043	-15.9872	-1.59764	4.593058	-3.33938

40.	*pct* callus 5	9.971535	-14.5735	-1.18445	4.569507	-4.21722
41.	*pct* regenerated shoot 1	1.583083	3.794905	9.627235	7.795511	1.987531
42.	*pct* regenerated shoot 2	-0.19169	2.542791	10.44703	8.121632	2.322484
43.	*pct* regenerated shoot 3	-0.47287	3.009223	10.27859	7.970128	2.01426
44.	*pct* regenerated shoot 4	-1.09999	4.622435	8.069116	5.049344	3.506419
45.	*pct* regenerated shoot 5	6.750509	2.044509	6.710744	1.114017	-4.63123
