# Supplementary material for: Metabolomic homeostasis shifts after callus formation and shoot regeneration in tomato
Source: PLoS One. 2017 May 8;12(5):e0176978. doi: 10.1371/journal.pone.0176978 (PMC5421760; doi:10.1371/journal.pone.0176978)
Supplement: S4 Table — (PDF) [file pone.0176978.s004.pdf]

**S4Table**: List of metabolites and its loading score for PC1 and PC2 of three stages viz. cotyledon, callus and regenerated shoot for WT, *shr* and *pct1-2* in combined, used for making collective PCA graph (Fig.2) Values were obtained using statistical analysis software (MS-DIAL) (37).

| S.No. | Metabolites | PC1 | PC2 |
|---|---|---|---|
| 1. | Erythritol | -0.021988812 | 0.00394531 |
| 2. | Ribose | 0.003597147 | -0.001715208 |
| 3. | L-Glycerol-2-phosphate | 0.000455114 | 0.000137687 |
| 4. | D-Psicopyranose | 0.018844629 | 0.006137419 |
| 5. | Talofuranose | 0.003508932 | -0.00197034 |
| 6. | Tagatose{BP} | 0.430961073 | -0.258332334 |
| 7. | Sorbose | -0.000120666 | 0.061661532 |
| 8. | Psicose | 0.237257613 | -0.098309687 |
| 9. | Fructose | 0.345304637 | -0.160505075 |
| 10. | Talopyranose | 0.091040044 | -0.060884831 |
| 11. | D-Glucose (Z) | 0.174007959 | -0.085841356 |
| 12. | Galactose | 0.037926129 | -0.040814792 |
| 13. | Talose | 0.015370875 | -0.015524453 |
| 14. | Glucose | 0.082584177 | -0.084870323 |
| 15. | 1-Methyl-α-D-glucopyranoside | 0.061206307 | -0.211619126 |
| 16. | β-D-Glucopyranose | 0.120735058 | -0.087469131 |
| 17. | Galactonate | 0.004872665 | -0.002439285 |
| 18. | myo-Inositol | 0.10533264 | -0.019407242 |
| 19. | D-Glucopyranose | 0.23770279 | 0.360770202 |
| 20. | 1-Methyl-β-D-galactopyranoside | 0.001393003 | 0.007823691 |
| 21. | Galactosylglycerol | 0.00214206 | -0.001246753 |
| 22. | Glucose-6-phosphate | 0.000896591 | -0.001920598 |
| 23. | myo-Inositol-2-P | 0.001104671 | 1.71387E-06 |
| 24. | Maltitol | 0.000381989 | -0.000284108 |
| 25. | 1,2,3-Butantriol | 0.001678692 | -0.000549977 |
| 26. | Cellobiose(isomer 1) | -6.36838E-05 | 0.000203459 |
| 27. | Sucrose | 0.174563216 | -0.024248043 |
| 28. | Turanose (isomer 1) | 0.036478455 | -0.128134589 |
| 29. | Cellobiose (isomer 2) | 0.001027342 | -0.00377468 |
| 30. | Turanose (isomer 2) | 0.001393747 | -0.004885578 |
| 31. | Trehalose | 0.001796729 | -0.000592215 |
| 32. | α-D-Galactopyranosyl α-(1,4)-D-galactopyranoside | 0.0030355 | -0.001785158 |
| 33. | Lactose | 0.001814773 | 0.000378934 |
| 34. | Isomaltose | 0.000827571 | -0.000374746 |
| 35. | Valine | 0.02010162 | 0.000822121 |
| 36. | Alanine | 0.030120455 | -0.015572015 |
| 37. | Serine | 0.011564951 | 0.004854238 |
| 38. | Threonine | 0.007702392 | 0.002126332 |
| 39. | Isoleucine | 0.009568701 | -0.000635509 |
| 40. | Proline | 0.011370694 | 0.005809423 |

| | | | |
|---|---|---|---|
| 41. | Glycine | 0.005172447 | -0.006049497 |
| 42. | Aspartate | 0.006075547 | -0.006567806 |
| 43. | Threonate | 0.003779486 | 0.00308947 |
| 44. | Glutamate | 0.008420334 | 0.004244251 |
| 45. | Phenylalanine | 0.002519787 | 0.000367693 |
| 46. | Asparagine | 0.077747807 | 0.068427955 |
| 47. | Glutamine | 0.298215401 | 0.219225938 |
| 48. | Lysine | 0.007744907 | 0.001517897 |
| 49. | Allantoin | 0.001266105 | 0.002075365 |
| 50. | Pentonate | 0.00141235 | -0.000462917 |
| 51. | Lyxonate | 0.001997474 | 1.99032E-05 |
| 52. | Gluconate | 0.004355779 | 0.000132681 |
| 53. | Palimitate | 0.018559915 | 0.004695722 |
| 54. | Glucarate | 0.00499326 | 0.004827302 |
| 55. | Galactarate | 0.002959364 | -0.006567306 |
| 56. | Linoleate | 0.000817666 | -9.5354E-05 |
| 57. | Stearate | 0.029125828 | 0.004159578 |
| 58. | Sinapinate | -0.000385502 | 0.000996568 |
| 59. | Caffeoylquinate | 0.004215301 | 0.007055412 |
| 60. | Ethanolamine | 0.016356099 | -0.005372545 |
| 61. | Ethanolamine P | 0.001154536 | 0.000857501 |
| 62. | Dopamine | 0.005461726 | 0.007948295 |
| 63. | Normetadrenaline | 0.002380351 | -0.00362003 |
| 64. | Noradrenaline | 0.003405748 | 0.002647377 |
| 65. | 5-Hydroxytryptamine | 0.000893204 | 0.001804563 |
| 66. | Pyridine, 2-hydroxy | 0.001825056 | -0.000228644 |
| 67. | Uridine | 0.000290878 | -0.000474817 |
| 68. | Adenosine | 0.000607212 | -0.000545296 |
| 69. | Borate | 0.018098254 | -0.008671463 |
| 70. | Pyruvate isomer 1 | 0.000529748 | 0.000286592 |
| 71. | 1,3-Propanediol | 0.011211544 | -0.010423863 |
| 72. | Lactate | 0.017572216 | -0.069266416 |
| 73. | Glycolate | 0.001127669 | -0.000574924 |
| 74. | Pyruvate isomer 2 | 0.000764467 | -0.000337921 |
| 75. | Oxalate | 0.002615083 | 0.000168587 |
| 76. | (R)-3-Hydroxybutyric acid | 0.000807576 | -0.005618908 |
| 77. | Phosphorate | 0.037456897 | -0.007130139 |
| 78. | Succinate | 0.005281934 | -0.005160543 |
| 79. | Glycerate | 0.001016945 | -0.001025498 |
| 80. | 3-Methyl-2-ketopiperazine | -0.013529207 | 0.002653532 |
| 81. | Pipecolate | -0.028350719 | 0.005560714 |
| 82. | Malate, 2-methyl- | 0.000243341 | 0.00029703 |
| 83. | Malate | 0.084281614 | -0.362179665 |
| 84. | 5-Oxoproline | 0.552913152 | 0.462211353 |
| 85. | GABA | 0.239742253 | -0.517908412 |
| 86. | Erythronate | 0.000418662 | 0.000710661 |
| 87. | 2-Imidazolidone-4-carboxylic acid | 0.002074663 | 0.000768955 |

| | | | |
|---|---|---|---|
| 88. | Pentanedioic acid | 0.001059286 | 7.44259E-05 |
| 89. | Citrate | 0.031455923 | -0.045284934 |
| 90. | Quinate | 0.006819638 | 0.006567098 |
| 91. | Caffeate | -0.002687469 | 0.000479218 |
