# Supplementary material for: Metabolomic homeostasis shifts after callus formation and shoot regeneration in tomato
Source: PLoS One. 2017 May 8;12(5):e0176978. doi: 10.1371/journal.pone.0176978 (PMC5421760; doi:10.1371/journal.pone.0176978)
Supplement: S5 Table — (PDF) [file pone.0176978.s005.pdf]

S5 Table: List of the metabolites and their relative levels in cotyledon (**Co**), callus (**Ca**) and in regenerated shoot (**RS**) of *shr* and *pct1*-2 mutants in comparison to WT tissue/organ of respective stage. Values in bold and in yellow color indicates significant changes with *p* ≤0.05. The fold changes are calculated as Log2 ratios. RI, Metabolite detection based on the retention index; **SD**, Metabolite detection based on standards. For each metabolite the KEGG ID is written in the extreme right column of table.

**Stages:** / **Metabolites:**	***shr* Co/ WT Co**	***shr* Ca/ WT Ca**	***shr* RS/ WT RS**	***pct1-2* Co/ WT Co**	***pct1-2* Ca/ WT Ca**	***pct1-2* RS/ WT RS**	**Method of Detection**	**KEGG ID**
Erythritol	**-1.007**	9.306	7.127	-1.940	8.951	10.557	RI	**C00503**
Ribose	**1.307**	0.120	-0.506	0.032	**0.356**	0.367		**C00121**
L-Glycerol-2-phosphate	**-1.354**	-0.263	0.187	-2.229	**-0.900**	-0.971	RI	**C03189**
D-Psicopyranose	-0.568	0.119	-0.052	-0.923	**-1.079**	-0.757	RI	**C06468**
Talofuranose	**-0.420**	0.304	0.321	**-0.966**	0.224	**-12.501**	RI	**Not found**
Tagatose{BP}	**-0.135**	0.341	0.161	**-0.566**	0.427	**-2.074**	RI	**D09007**
Sorbose	**-1.334**	0.492	0.210	**-2.184**	-0.069	**18.389**	RI	**C00247**
Psicose	**-1.311**	0.161	0.056	-1.046	**0.269**	-0.040	RI	**C06468**
Fructose	**-1.411**	0.265	0.132	**-2.291**	**0.326**	0.113	SD	**Not found**
Talopyranose	**-1.358**	-0.056	0.210	**-2.280**	0.218	-0.138	RI	**Not found**
D-Glucose (Z)	-2.042	0.095	0.123	-2.940	**0.311**	-0.073	SD	**C00221**
Galactose	**-1.190**	0.132	-0.134	**-2.498**	0.988	0.172	SD	**C00124**
Talose	**-1.619**	-0.161	-0.371	**-2.526**	0.662	0.187	RI	**C06467**
Glucose	**-1.346**	-0.101	-0.328	**-2.250**	**0.781**	0.239	SD	**C00031**
1-Methyl-α-D-glucopyranoside	-4.026	0.178	0.181	-4.979	**3.363**	**2.293**	RI	**C00388**
Glucopyranose isomer1	**-2.082**	0.083	0.214	**-8.094**	0.402	-0.150	RI	**C00221**
Galactonate	**1.375**	0.869	0.225	**-5.098**	**1.285**	**0.645**	RI	**C00880**
myo-Inositol	0.772	0.514	**0.846**	-1.477	**0.627**	**1.016**	RI	**C00137,D08079, C01204,C06153, D01813**
Glucopyranose isomer2	**2.682**	**6.464**	**5.412**	-1.793	**0.676**	**5.562**	RI	**C00031**
1-Methyl-β-D-galactopyranoside	**1.022**	**9.361**	**13.436**	**-1.068**	**-3.702**	**14.408**	RI	**C03619**
Galactosylglycerol	-0.174	**-0.922**	**-0.640**	**-4.127**	-0.102	-0.333	RI	**C05401**
Glucose-6-phosphate	-0.011	-0.120	**-1.001**	**-1.319**	**0.632**	-0.035	RI	**C00668,C00092, C02965**
myo-Inositol-2-phosphate	-0.955	-0.153	0.083	-3.121	-0.241	-0.539	RI	**C00137**
Maltitol	0.245	**1.886**	**-0.934**	-0.276	1.734	-1.233	RI	**D04845**

Stages: / Metabolites:	*shr* Co/ WT Co	*shr* Ca/ WT Ca	*shr* RS/ WT RS	*pct1-2* Co/ WT Co	*pct1-2* Ca/ WT Ca	*pct1-2* RS/ WT RS	Method of Detection	KEGG ID
1,2,3-Butantriol	0.555	**1.083**	0.047	-0.239	0.808	0.184	RI	**Not found**
Cellobiose(isomer 1)	-0.468	3.629	-1.009	**-1.983**	1.316	3.224	RI	**C06422,C00185**
Sucrose	3.751	0.114	0.221	**3.666**	0.008	-0.003	SD	**C00089**
Turanose (isomer 1)	**-0.418**	0.378	0.183	**-2.572**	**2.538**	16.585	RI	**C19636**
Cellobiose (isomer 2)	**-1.274**	0.105	-7.093	**-2.229**	1.839	5.575	RI	**C06422**
Turanose (isomer 2)	1.928	-10.49	0.222	**1.798**	1.430	13.745	RI	**C19636**
Trehalose	-8.720	0.340	-0.399	1.193	0.635	-0.614	RI	**C01083**
α-D-Galactopyranosyl α-(1,4)-D-galactopyranoside	**-1.933**	0.326	-0.091	**-2.211**	1.111	0.975	RI	**Not found**
Lactose	**-1.672**	0.761	-0.367	**-2.490**	1.357	2.259	RI	**C01970**
Isomaltose	**-1.293**	-0.117	**-0.890**	**-2.189**	0.228	1.422	RI	**C00252**
Valine	-1.199	**1.315**	0.199	-3.029	**0.557**	0.528	RI	**C00183**
Alanine	0.948	0.165	**0.523**	**-2.193**	**0.674**	-0.497	RI	**C00041**
Serine	-1.045	0.199	**0.393**	-6.024	**-0.450**	0.235	RI	**C00065**
Threonine	0.070	0.813	**0.412**	-2.121	**0.208**	0.201	RI	**C00188**
Isoleucine	**-1.278**	**1.400**	0.161	-2.250	**0.955**	0.177	RI	**C00407**
Proline	-2.473	1.241	**1.705**	-0.479	**0.553**	1.163	RI	**C00148,D00035, C00763**
Glycine	0.468	0.358	0.148	-1.208	**1.190**	-0.021	RI	**C00037**
Aspartate	**-1.341**	0.638	0.296	**-2.324**	**0.816**	0.051	RI	**C00049,D00013**
Threonate	-1.408	2.038	3.319	-2.155	0.600	4.668	RI	**C01620**
Glutamate	**1.758**	1.000	-0.201	**-0.524**	-0.069	-1.988	RI	**C00025**
Phenylalanine	**-1.417**	0.191	-0.132	**-2.249**	-0.117	-0.837	RI	**C02057**
Asparagine	0.271	1.485	0.571	**0.888**	**-0.800**	-0.886	RI	**C00152**
Glutamine	-0.400	0.223	0.190	-0.247	**-1.854**	-2.091	RI	**C00064**
Lysine	0.311	**2.389**	**1.372**	0.748	**0.997**	1.648	RI	**C00047**
Allantoin	1.984	-0.951	0.340	-0.428	**-10.490**	-0.182	RI	**C01551**
Pentonate	**-1.449**	-1.824	-0.233	**-2.302**	-0.193	-0.312	RI	**Not found**
Lyxonate	**-1.311**	**0.785**	0.173	**-2.151**	0.339	0.593	RI	**Not found**
Gluconate	**-1.094**	-0.022	**-0.842**	**-2.429**	0.339	0.775	RI	**C00257**
Palimitate	**-1.638**	-0.115	0.580	**-2.494**	-0.082	0.139	RI	**C00249,D05341**
Glucarate	-0.253	**1.387**	**0.380**	**-1.938**	**1.248**	1.049	RI	**C00818**
Galactarate	-0.959	0.522	0.076	**-2.027**	**2.006**	0.041	RI	**C00879**
Linoleate	**-1.323**	-0.187	-0.409	**-2.130**	-0.017	-0.815	RI	**C01595**
Stearate	**0.601**	-0.333	**0.575**	**-1.482**	-0.133	0.421	RI	**C01530**
Sinapinate	**1.338**	4.969	**2.708**	**-0.749**	**2.393**	3.326	RI	**C00482**

**Stages:** / **Metabolites:**	***shr* Co/ WT Co**	***shr* Ca/ WT Ca**	***shr* RS/ WT RS**	***pct1-2* Co/ WT Co**	***pct1-2* Ca/ WT Ca**	***pct1-2* RS/ WT RS**	**Method of Detection**	**KEGG ID**
Caffeoylquinate	-0.594	10.909	0.002	-0.123	9.876	0.298	RI	**Not found**
Ethanolamine	2.460	0.610	0.096	0.598	**0.564**	0.162	RI	**C00189,D05074**
Ethanolaminephosphate	**-1.311**	**0.850**	0.460	**-2.250**	-1.766	-10.635	RI	**C14535**
Dopamine	**-0.917**	**0.709**	**0.810**	**-2.865**	0.223	2.111	RI	**C03758,D07870**
Normetadrenaline	-1.616	**1.644**	0.145	-1.980	**1.988**	0.696	RI	**Not found**
Noradrenaline	-0.869	**2.013**	**0.528**	**-2.254**	**0.612**	1.330	RI	**C00547,D00076**
5-Hydroxytryptamine	**-1.293**	**1.529**	**3.284**	**-2.149**	**-1.322**	2.564	RI	**C00780**
Pyridine, 2-hydroxy	**-1.365**	0.021	0.123	**-1.775**	0.267	1.008	RI	**C00747**
Uridine	**-1.272**	-0.524	-0.178	-0.594	1.357	-1.008	RI	**C00299**
Adenosine	-0.261	0.183	**0.453**	**-1.549**	0.661	0.184	RI	**C00212**
Borate	-1.506	-0.028	0.414	-0.651	0.234	0.529	RI	**C12486**
Pyruvate isomer 1	**-1.311**	**1.154**	-0.045	**-2.292**	0.791	2.543	RI	**C00022**
1,3-Propanediol	**-1.280**	-0.271	-0.028	**-0.990**	0.625	0.068	RI	**C02457**
Lactate	0.377	-0.304	0.291	-7.427	3.240	1.127	RI	**C01432**
Glycolate	**-5.824**	0.291	0.372	**-2.257**	1.513	0.127	RI	**C00160**
Pyruvate isomer 2	-6.627	**0.619**	0.469	-7.541	0.691	0.659	RI	**C00022**
Oxalate	-6.174	-0.111	-0.008	-7.014	-0.374	-0.623	RI	**C00209**
(R)-3-Hydroxybutyric acid	**7.754**	**1.132**	**7.262**	6.167	6.026	8.606	RI	**C01089**
Phosphorate	**-1.320**	**0.871**	0.435	**-2.200**	0.620	-0.238	RI	**C00009,D05467**
Succinate	**-1.359**	**0.898**	0.175	**-2.199**	1.644	0.094	RI	**C00042**
Glycerate	0.226	**1.120**	0.229	**-2.213**	1.680	0.007	RI	**C00258**
3-Methyl-2-ketopiperazine	**0.708**	0.397	0.345	**-1.315**	0.013	-0.053	RI	**Not found**
Pipecolate	**0.899**	2.367	0.363	**-0.878**	1.441	3.461	RI	**Not found**
Malate, 2-methyl-	**-1.468**	-0.302	0.179	**-2.292**	-8.725	1.145	RI	**C00711,C03668, D04843**
Malate	-4.087	14.943	16.190	-1.358	18.184	17.199	RI	**C00711**
5-Oxoproline	1.543	-0.270	0.408	-3.058	-1.292	-0.076	RI	**C01879**
GABA	1.493	**-0.374**	0.001	-1.247	1.280	-0.469	RI	**C00334**
Erythronate	-1.286	**4.272**	2.451	2.646	-5.548	4.260	RI	**Not found**
2-Imidazolidone-4-carboxylic acid	1.513	**0.597**	**0.636**	**-2.268**	-0.601	-0.096	RI	**Not found**
Pentanedioic acid	-1.468	0.472	**0.423**	-2.336	0.080	0.136	RI	**C00489**
Citrate	**-1.319**	**-0.425**	-0.581	**-2.291**	1.226	-0.885	RI	**C00158,D00037**
Quinate	**-0.695**	**3.471**	2.323	**-2.193**	2.534	3.962	RI	**Not found**
Caffeate	**-0.949**	4.617	6.072	**-3.057**	6.204	6.875	RI	**C01197,C01481**
