# Supplementary figures and images for: Metabolomic homeostasis shifts after callus formation and shoot regeneration in tomato
Source: PLoS One. 2017 May 8;12(5):e0176978. doi: 10.1371/journal.pone.0176978 (PMC5421760; doi:10.1371/journal.pone.0176978)

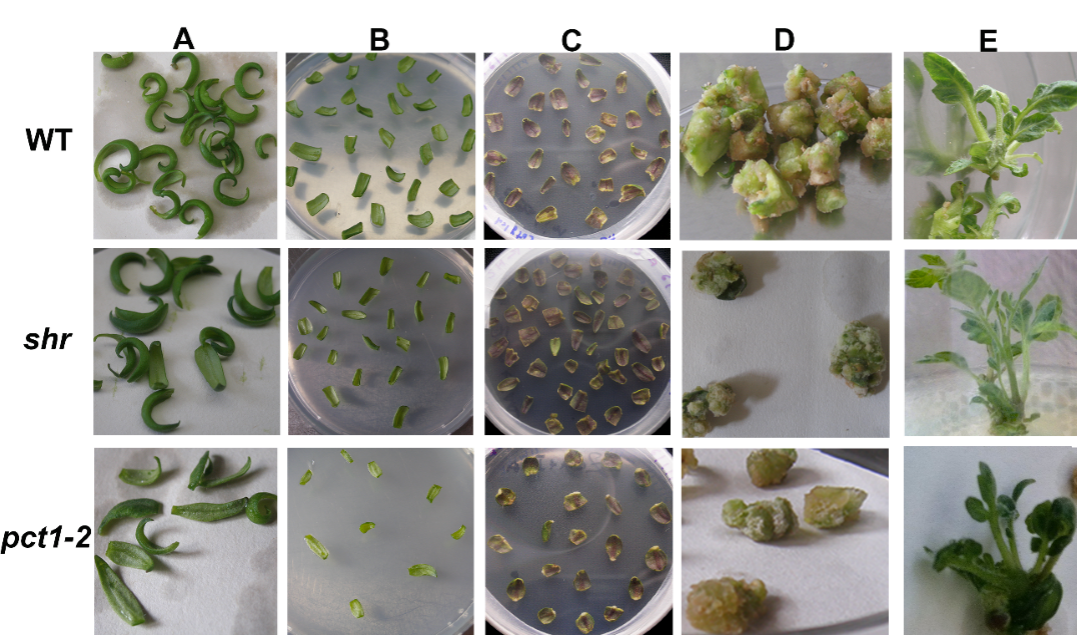

Supplement: S1 Fig — (A) Phenotype of 9–10 days old cotyledons used as explants. (B) Trimmed cotyledons placed on CIM (C) Initiation of callus after 20–25 days of incubation on cotyledon margin (D) Callus after second subculture on SIM (E) Differentiated tissue. (TIF) [file pone.0176978.s009.tif]

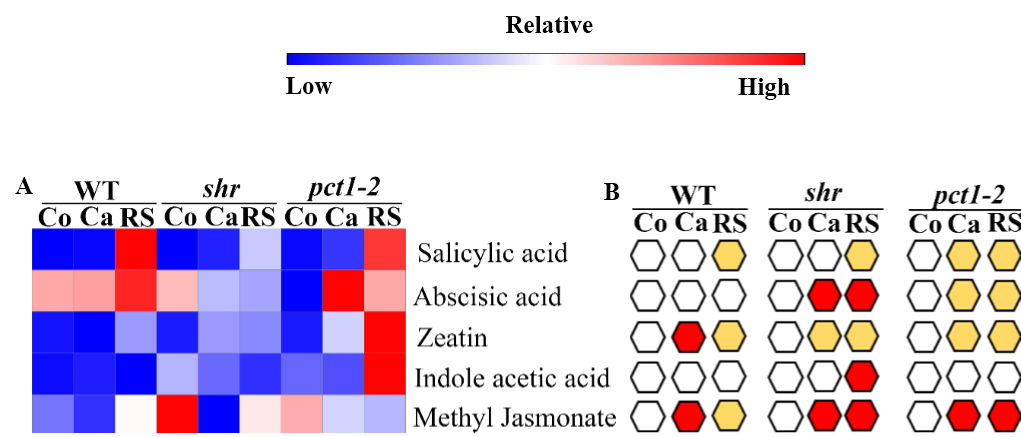

Supplement: S2 Fig — The relative levels are indicated by varying color intensity (low-blue, high-red) with reference to respective cotyledons. The coloured hexagons on right of heat map represent the statistically significant (p ≤0.05) upregulation (yellow) or downregulation (red) of respective hormones. (TIF) [file pone.0176978.s010.tif]

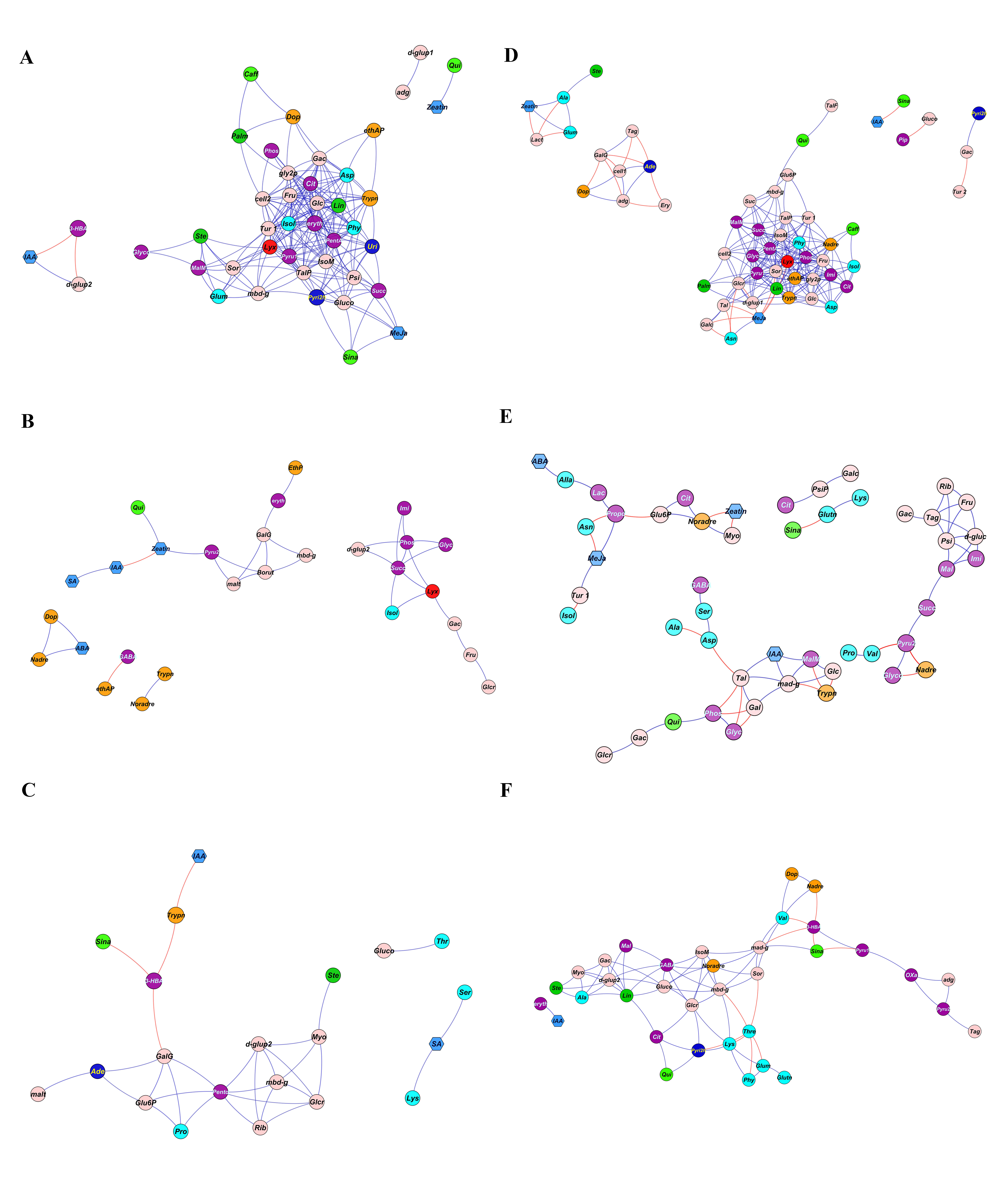

Supplement: S5 Fig — Networks were plotted using Cytoscape with sugars (light pink), amino acids (blue), organic acids (purple), fatty acids (dark green), monoamines (golden yellow), nucleotides (dark blue), hydroxycinnamic acids (grass green) and phytohormones (blue hexagon). Only interactions (p ≤0.05) with r ≥ ±0.9 were used for generating the network. The blue and red lines between the different nodes indicate positive and negative correlations respectively. The significant changes in metabolites and hormones were calculated by determining shr/WT and pct1-2/WT ratio of individual metabolites for each stage. The full names of the metabolites depicted on the network are given in supplementary Excel File (S7 Table). (TIF) [file pone.0176978.s013.tif]

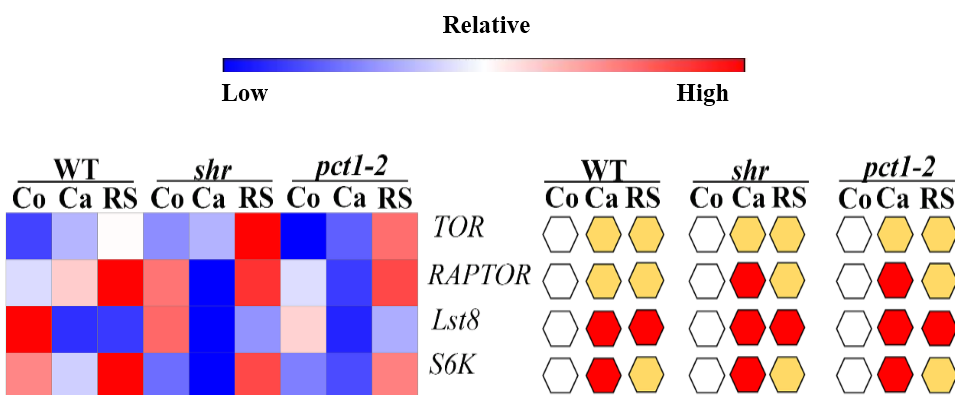

Supplement: S6 Fig — The relative levels are indicated by varying color intensity (low-blue, high-red) with reference to respective cotyledons. The coloured hexagons on right of heat map represent the statistically significant (p ≤0.05) upregulation (yellow) or downregulation (red) of respective genes. (TIF) [file pone.0176978.s014.tif]
