# Supplementary material for: Metabolomic homeostasis shifts after callus formation and shoot regeneration in tomato
Source: PLoS One. 2017 May 8;12(5):e0176978. doi: 10.1371/journal.pone.0176978 (PMC5421760; doi:10.1371/journal.pone.0176978)
Supplement: S3 Fig — Networks were divided into independent clusters using Cytoscape. Metabolites not mapping in any cluster are present as independent entities. Networks were plotted using Cytoscape with sugars (light pink), amino acids (sea green), organic acids (purple), sugar acid (red) fatty acids (dark/lime green), monoamines (dark golden), nucleotides (dark blue), hydroxycinnamic acids (lawn green) and phytohormones (blue hexagon). Only interactions (p ≤0.05) with r ≥ ±0.9 were used for generating the network. The blue and red lines between the different nodes indicate positive and negative correlations respectively. (PDF) [file pone.0176978.s011.pdf]

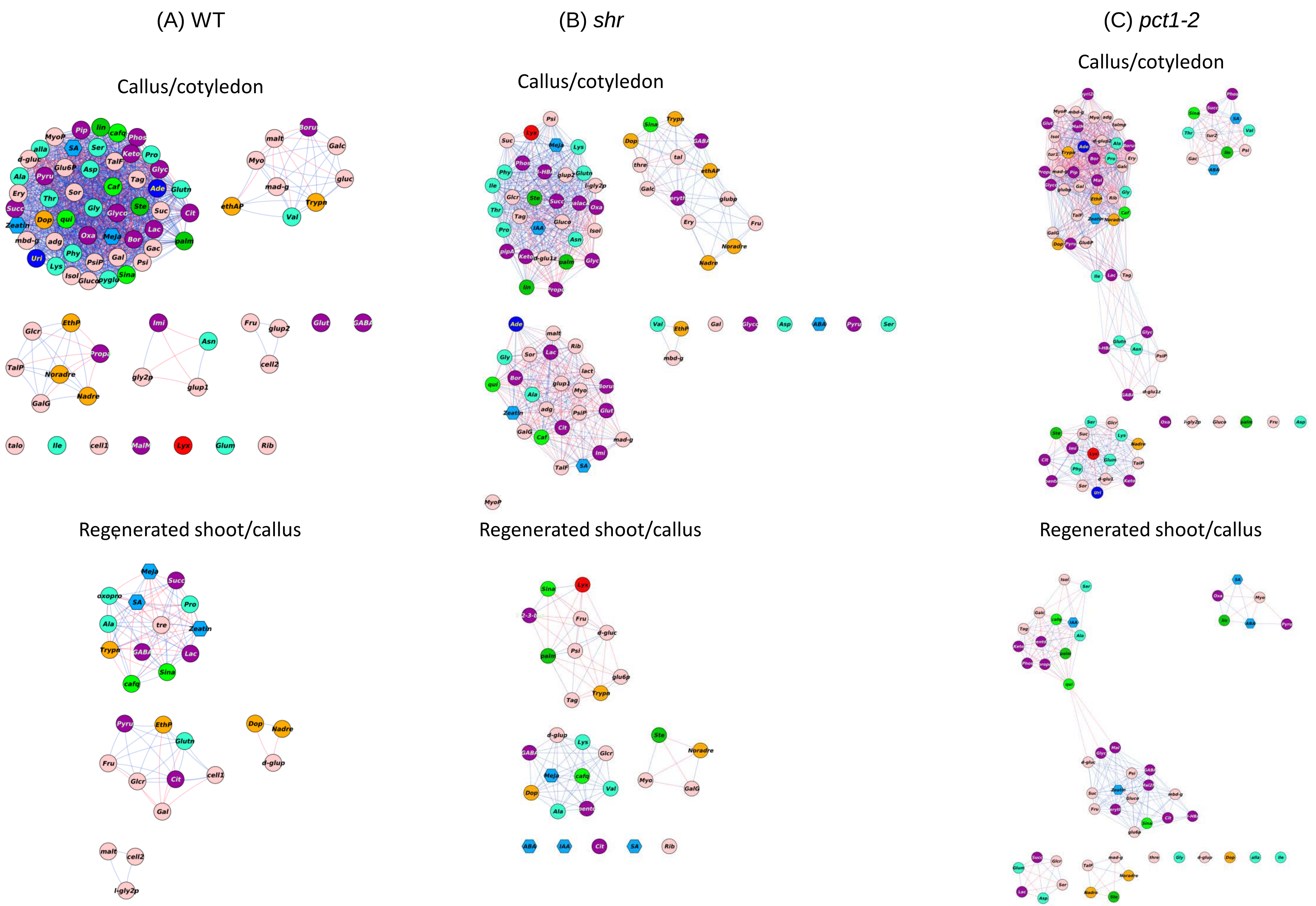
(A) WT
Callus/cotyledon
Regenerated shoot/callus
(B) shr
Callus/cotyledon
Regenerated shoot/callus
(C) pct1-2
Callus/cotyledon
Regenerated shoot/callus
