# Supplementary material for: Metabolomic homeostasis shifts after callus formation and shoot regeneration in tomato
Source: PLoS One. 2017 May 8;12(5):e0176978. doi: 10.1371/journal.pone.0176978 (PMC5421760; doi:10.1371/journal.pone.0176978)
Supplement: S4 Fig — The clustering significantly changes during transition from cotyledon to callus and callus to regenerated shoots of WT (A), shr (B) and pct1-2 (C) respectively. Metabolites and hormones grouping in independent clusters were colored with different colors. In each figure the color code of cluster is mentioned. (PDF) [file pone.0176978.s012.pdf]

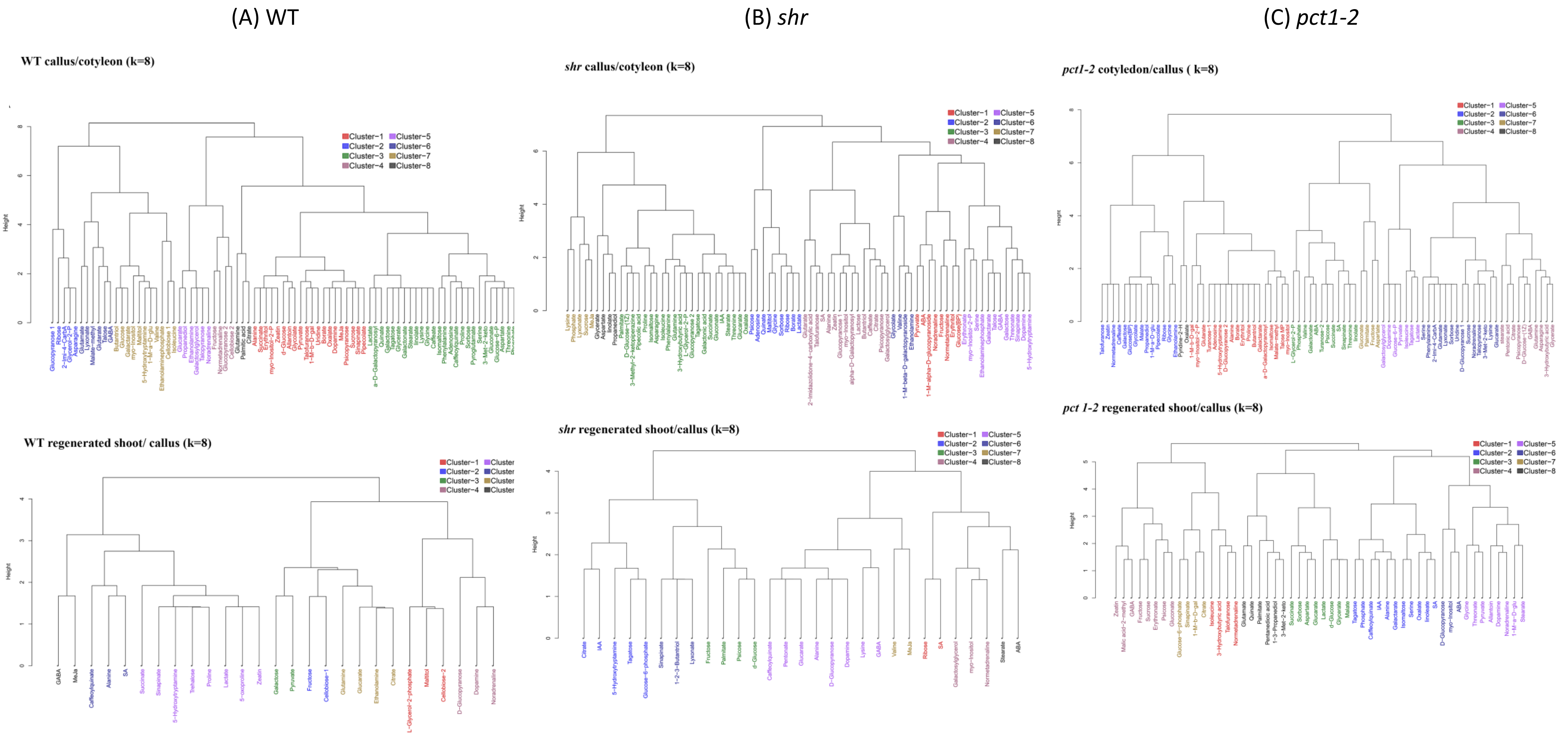
(A) WT
(B) shr
(C) pct1-2
WT callus/cotyleon (k=8)
shr callus/cotyleon (k=8)
pct1-2 cotyledon/callus (k=8)
WT regenerated shoot/ callus (k=8)
shr regenerated shoot/callus (k=8)
pct 1-2 regenerated shoot/callus (k=8)
Height
Cluster−1
Cluster−2
Cluster−3
Cluster−4
Cluster−5
Cluster−6
Cluster−7
Cluster−8
